# Inflammasome Sensor NLRP1 Controls Rat Macrophage Susceptibility to *Toxoplasma gondii*

**DOI:** 10.1371/journal.ppat.1003927

**Published:** 2014-03-13

**Authors:** Kimberly M. Cirelli, Gezahegn Gorfu, Musa A. Hassan, Morton Printz, Devorah Crown, Stephen H. Leppla, Michael E. Grigg, Jeroen P. J. Saeij, Mahtab Moayeri

**Affiliations:** 1 Massachusetts Institute of Technology, Department of Biology, Cambridge, Massachusetts, United States of America; 2 Molecular Parasitology Section, Laboratory of Parasitic Diseases, NIAID, NIH, Bethesda, Maryland, United States of America; 3 Department of Pharmacology, University of California-San Diego, La Jolla, California, United States of America; 4 Microbial Pathogenesis Section, Laboratory of Parasitic Diseases, NIAID, NIH, Bethesda, Maryland, United States of America; University of Massachusetts, United States of America

## Abstract

*Toxoplasma gondii* is an intracellular parasite that infects a wide range of warm-blooded species. Rats vary in their susceptibility to this parasite. The *Toxo1* locus conferring *Toxoplasma* resistance in rats was previously mapped to a region of chromosome 10 containing *Nlrp1*. This gene encodes an inflammasome sensor controlling macrophage sensitivity to anthrax lethal toxin (LT) induced rapid cell death (pyroptosis). We show here that rat strain differences in *Toxoplasma* infected macrophage sensitivity to pyroptosis, IL-1β/IL-18 processing, and inhibition of parasite proliferation are perfectly correlated with NLRP1 sequence, while inversely correlated with sensitivity to anthrax LT-induced cell death. Using recombinant inbred rats, SNP analyses and whole transcriptome gene expression studies, we narrowed the candidate genes for control of *Toxoplasma*-mediated rat macrophage pyroptosis to four genes, one of which was *Nlrp1*. Knockdown of *Nlrp1* in pyroptosis-sensitive macrophages resulted in higher parasite replication and protection from cell death. Reciprocally, overexpression of the NLRP1 variant from *Toxoplasma*-sensitive macrophages in pyroptosis-resistant cells led to sensitization of these resistant macrophages. Our findings reveal *Toxoplasma* as a novel activator of the NLRP1 inflammasome in rat macrophages.

## Author Summary

Inflammasomes are multiprotein complexes that are a major component of the innate immune system. They contain “sensor” proteins that are responsible for detecting various microbial and environmental danger signals and function by activating caspase-1, an enzyme that mediates cleavage and release of the pro-inflammatory cytokines, IL-1β and IL-18. *Toxoplasma gondii* is a highly successful protozoan parasite capable of infecting a wide range of host species that have variable levels of resistance. Rat strains have been previously shown to vary in their susceptibility to this parasite. We report here that rat macrophages from different inbred strains also vary in sensitivity to *Toxoplasma* induced lysis. We find that NLRP1, an inflammasome sensor whose only known agonist is anthrax LT, is also activated by *Toxoplasma* infection. In rats there is a perfect correlation between NLRP1 sequence and macrophage sensitivity to *Toxoplasma*-induced rapid cell death, inhibition of parasite proliferation, and IL-1β/IL-18 processing. *Nlrp1* genes from sensitive rat macrophages can confer sensitivity to this rapid cell death when expressed in *Toxoplasma* resistant rat macrophages. Our findings suggest *Toxoplasma* is a new activator of the NLRP1 inflammasome.

## Introduction

*Toxoplasma gondii* is an obligate intracellular parasite, for which different host species or strains within a species display variable susceptibilities. Different *Toxoplasma* strains also differ in virulence within the same host, suggesting variation in effectors among parasite strains and/or their impact in various hosts. Host innate immunity is known to play a critical role in susceptibility to infection. In mice, for example, resistance to *Toxoplasma* infection is critically dependent on the induction of IL-12, which subsequently induces IFN-γ, the main mediator of toxoplasmicidal activities (for review, see [1]).

Rats, like humans, are quite resistant to *Toxoplasma* infection when compared to mice. However varying levels of resistance also exist among rat strains. The resistance of the Lewis (LEW) strain is characterized by total clearance of the parasite, failure to develop cysts and the absence of a strong antibody response. Fischer (CDF) and Brown Norway (BN) rats, however, are susceptible to chronic infection and develop transmissible cysts in their brain and muscle tissue [2], [3]. Resistance in rats is a dominant trait and is linked to myeloid cell control of parasite proliferation [2], [3].

Linkage analyses of LEWxBN F2 progeny was previously used to map *Toxoplasma* resistance in rats to a single genetic locus, termed *Toxo1*, within a 1.7-cM region of chromosome 10 [2]. We noted that this locus overlaps with the locus that controls rat and macrophage sensitivity to the anthrax lethal toxin (LT) protease. Inbred rat strains and their macrophages exhibit a perfectly dichotomous phenotype in response to LT: animals either die rapidly (<1 h) or exhibit complete resistance to the toxin [4]. Only macrophages from LT-sensitive rat strains undergo rapid caspase-1 dependent death (pyroptosis). The HXB/BXH recombinant inbred (RI) rat collection, developed from the SHR/Ola and BN-Lx congenic parental strains [5]–[7], with opposing LT sensitivities, was used to map anthrax toxin susceptibility to a single locus at 55.8–58.1 Mb of rat chromosome 10. SNP analyses and sequence correlation to phenotype implicated the inflammasome sensor *Nlrp1* (nucleotide-binding oligomerization domain, leucine-rich repeat protein 1) as the likely susceptibility locus. NLRP1 is a member of the NLR cytosolic family of pathogen-associated molecular pattern molecule (PAMP) sensors, the activation of which leads to recruitment and autoproteolytic activation of caspase-1, followed by cleavage and release of the proinflammatory cytokines IL-1β and IL-18. NLR-mediated activation of caspase-1 is typically accompanied by rapid death of macrophages through a process known as pyroptosis (for review see [8], [9]). NLRP1 sequences from 12 inbred rat strains show a perfect correlation between sensitivity and the presence of an N-terminal eight amino acid (aa) LT cleavage site [4], [10]. Proteolytic cleavage by LT activates the NLRP1 inflammasome in rat macrophages leading to rapid caspase-1 dependent cell death (pyroptosis) and cytokine processing [10].

We hypothesized that the *Toxo1* locus could be *Nlrp1* as the macrophage is an important carrier of the parasite [11], [12] and inflammasome-mediated pyroptosis of this cell could impact *in vivo* parasite dissemination. The recent association of polymorphisms in the human *NLRP1* gene with susceptibility to congenital toxoplasmosis, evidence that P2X(7) receptors influence parasite proliferation in mouse cells, and the finding that IL-1β responses in *Toxoplasma* infected human monocytes are dependent on caspase-1 and the inflammasome adaptor protein ASC all suggest that the inflammasome plays a role in determining the outcome of *Toxoplasma* infection in humans and mice [13]–[15].

Our results indicate that rat strain macrophages exhibit dichotomous susceptibilities to *Toxoplasma*-induced rapid lysis and associated cytokine processing in a manner correlated with NLRP1 sequence. We go on to show that *Nlrp1* knockdown in *Toxoplasma*-sensitive macrophages protects against this cell death while overexpression of certain variants of the gene in resistant macrophages can sensitize these cells to the parasite-induced pyroptosis. Our findings establish *Toxoplasma* as the second known activator of the inflammasome sensor NLRP1 and suggest a mechanism of host resistance involving activation of this sensor.

## Materials and Methods

### Ethics statement

All animal experiments were performed in strict accordance with guidelines from the NIH and the Animal Welfare Act, approved by the Animal Care and Use Committee of the National Institute of Allergy and Infectious Diseases, National Institutes of Health (approved protocols LPD-8E and LPD-22E) and the MIT Committee on Animal Care (assurance number A-3125-01).

### Materials

Ultra-pure lipopolysaccharide (LPS), nigericin (Calbiochem/EMD Biosciences, San Diego, CA and Invivogen, San Diego, CA), 3-(4,5-dimethyl-2-thiazolyl)-2,5-diphenyl tetrazolium bromide (MTT) (Sigma, St Louis, MO), Mycalolide B (Wako USA, Richmond, VA) were purchased. LT consists of two polypeptides, protective antigen (PA) and lethal factor (LF). Endotoxin-free LF and PA were purified from *B. anthracis* as previously described [16]. Concentrations of LT refer to equal concentrations of PA+ LF (ie, LT 1 µg/ml is LF+PA, each at 1 µg/ml).

### Rats

Brown Norway (BN/Crl; BN), Fischer CDF (F344/DuCrl; CDF), Lewis (LEW/Crl; LEW), Spontaneously Hypertensive Rat (SHR/NCrl; SHR) and Sprague Dawley (SD) rats (8–12 weeks old) were purchased from Charles River Laboratories (Wilmington, MA) and used as source of bone marrow. Certain experiments utilized F344/NTac rats from Taconic Farms (Germantown, NY). The recombinant inbred (RI) rat strains HXB1, HXB15 and HXB29 are derived from the progenitor strains BN-Lx and SHR/Ola [5]–[7]. The microsatellite marker genotypes and linkage maps used in mapping LT sensitivity using the HXB/BXH RI collection have been described [4].

### Parasites

Tachyzoites from Type I (RH) and Type II (76K or Prugniaud [PRU]) strains expressing luciferase and GFP from the plasmid pDHFR-Luc-GFP gene cassette [17] were used for most experiments. The following strains (haplogroup/type in parentheses) were used in a survey of effects on rat macrophages: GT1 (I), ME49 (II), DEG (II), CEP (III), VEG (III), CASTELLS (IV), MAS (IV), GUY-KOE (V), GUY-MAT (V), RUB (V), BOF (VI), GPHT(VI), CAST (VII), P89 (IX), GUY-DOS (X), VAND (X), Cougar (XI), RAY (XII), WTD3 (XII). All parasite strains were routinely passaged *in vitro* in monolayers of human foreskin fibroblasts (HFFs) at 37°C in the presence of 5% CO_2_ , spun and washed prior to quantification by hemocytometer counts. In some experiments, Mycalolide B (3 µM, 15 min) or DMSO was used to pretreat isolated parasites prior to washing in PBS (3×) before infections. The viability of these Mycalolide B- or DMSO-treated parasites was assessed in each experiment by adding them to a monolayer of HFFs and staining for STAT6 activation induced by the parasite secreted rhoptry kinase ROP16. Mycalolide B-treated parasites were able to secrete ROP16 but could no longer invade. In other experiments parasites were lysed using cell lysis solution (Abcam, Cambridge, MA) to assess LDH activity. Parasite viability and health differed from experiment to experiment, accounting for variations in experimental results that are reflected in standard deviations for pooled studies.

### Cell culture, nucleofection, toxicity, cytokine measurement, Western and microscopy studies

BMDMs were cultured in Dulbecco's modified Eagle medium (DMEM) supplemented with 30–33% L929 cell supernatants as previously described [18], [19], or with minor modification (20% fetal bovine serum, 50 µg/ml penicillin and 50 µg/ml streptomycin). NLRP1-expressing HT1080 or macrophage BMAJ lines and their growth conditions have been previously described [10]. The c-myc tagged rat caspase-1 gene was synthesized by GeneArt (Regensburg, Germany) and cloned into pcDNA(3.1)+ vector for expression in HT1080 cells by transfection with TurboFect (Fermentas, Glen Burnie, MD) using manufacturer's protocols. HA-tagged LEW and CDF NLRP1 expressing constructs used in BMDM nucleofection experiments have been described [10]. Endotoxin-free control vector or various NLRP1 expressing constructs were purified (Endofree kit, Qiagen, Germantown, MD) and nucleofected (1.2–3.0 µg/1×10^6^ cells/nucleofection) into rat BMDMs using the Amaxa Nucleofector (Lonza, Walkersville, MD) (kit VPA-1009, program Y-001). Nucleofections were performed at −24, −36, −48, and −72 h prior to infections with parasite. Toxicity and viability assays were modified from previously described methods [18], [19]. Briefly, animal-derived BMDMs with or without LPS priming 0.1 µg/ml, 1 h) were infected with *Toxoplasma* at various multiplicities of infection (MOIs) or treated with anthrax LT (1 µg/ml) and cell viability was assessed at different time points by one of three methods. 1) MTT staining (0.5 mg/ml) was performed as previously described [18], [19]; 2) MTS ([3-(4,5-dimethylthiazol-2-yl)-5-(3-carboxymethoxyphenyl)-2-(4-sulfophenyl)-2H-tetrazolium) was used to measure viability with the CellTiter 96 AQueous One Solution Cell Proliferation Assay (Promega, Madison, WI) according to manufacturer protocol ; 3) Lactate dehydrogenase (LDH) release assays were performed in select experiments according to manufacturer protocol (Roche Diagnostics, Mannheim, Germany). For luciferase assays, cells were lysed in 1× Lysis Reagent (Promega) and luciferin (Caliper Life Sciences, Hopkinton, MA) added prior to luciferase activity readings. In all experiments culture supernatants were removed for cytokine measurements by ELISA (R&D Systems, Minneapolis, MN and Abnova Corporation, Walnut, CA) or Western blotting, with or without concentration using Amicon filters (3000 Molecular weight cutoff) (Millipore, Billerica, MA). Cell lysates were made from infected cells as previously described [18], [19]. Anti-rat IL-1β (Abcam or Santa Cruz BT, Santa Cruz, CA), anti-rat IL-18 (Santa Cruz BT) or anti-HA antibody (Roche Diagnostics) were used as primary antibodies. Secondary IR-dye conjugated or HRP-conjugated antibodies were from Rockland (Gilbertsville, PA), Licor Biosciences (Lincoln, NE) or Jackson Immunoresearch (West Grove, PA). Immun-Star Western C substrate (BioRad, Hercules, CA) and a charge-coupled device camera (Chemidoc XRS, Biorad) or the Odyssey Infrared Imaging System (Licor Biosciences) was used for Western visualization depending on the secondary antibody used for detection. For select microscopy studies phase contrast images of MTT-stained cells were acquired on a Nikon Eclipse TE2000-U microscope without cell fixation followed by fluorescence image collection for the same field. For other fluorescence microscopy studies nucleofected cells were plated on poly-lysine (Sigma, St. Louis, MO) treated coverslips prior to infection and fixed (4% paraformaldyde, Electron Microscopy Sciences, Hatfield, PA), with or without permeabilization (0.1% TritonX-100). Immunostaining was with anti-HA antibody (Roche Diagnostics) and Alexa Fluor 594 secondary antibody (Invitrogen). For immunofluorescence staining of surface antigen (SAG)-1 or assessment of STAT6 phosphorylation, cells were fixed (3% formaldehyde) and permeabilized (0.2% TritonX-100 or 100% ethanol) followed by staining with a rabbit polyclonal antibody against human pSTAT6 (Santa Cruz BT, Santa Cruz, CA) or rabbit polyclonal antibody against *Toxoplasma* surface antigen (SAG)-1. Alexa Fluor 594 secondary antibodies were used for detection as has been described [20].

### RNA knockdown studies

NLRP1 knockdown was achieved by two methods. First, siGENOME SMARTpool siRNA set of four, targeting rat *Nlrp1a* (D-983968-17, D-983968-04, D-983968-03, D-983968-02; target sequences of GGUCUGAACAUAUAAGCGA, CCACGGUGUUCCAGACAAA, GCAUUACGUUCUCUCAUGU, GCAGUACGCAGUCUCUGUA) and siGENOME non-targeting siRNA pool (D-001206-14-05, target sequences of UAAGGCUAUGAAGAGAUAC, AUGUAUUGGCCUGUAUUAG, AUGAACGUGAAUUGCUCAA, UGGUUUACAUGUCGACUAA) were obtained from Thermo Sciences-Dharmacon (Pittburgh PA). siRNA pools were nucleofected (200 nM) into rat BMDMs (day 5 or 6 of differentiation) using the Amaxa Nucleofector (Lonza, Walkersville, MD) (kit VPA-1009, program Y-001) at −24, −36, −48, and −72 h prior to infection. Alternatively, on day 2 of differentiation BMDMs were infected with high-titer lentivirus (Broad Institute RNAi consortium) encoding shRNA against target sequence TGATCTACTATCGAGTCAATC designed against murine *Nlrp1b* with high homology (18 out of 21 nucleotides, perfect seed sequence identity) to rat *Nlrp1a* or the control shRNA with sequence (GCTTATGTCGAATGATAGCAA or GTCGGCTTACGGCGGTGATTT). Puromycin selection (6 µg/ml) of lentivirus infected cells, followed by qPCR analysis (*Nlrp1a* primers were 5′CATGTGATTTGGACCTGACG′3, 5′TCTTTGCCTGCAAGTTTCCT′3, actin primers were 5′GTCGTACCACTGGCATTGTG′3, 5′CTCTCAGCTGTGGTGGTGAA′3) verified knockdown. Expression of *Nlrp1a* was normalized against actin expression levels.

### Whole transcriptome sequencing and SNP analyses

SNP and haplotype analyses for the HXB, SHR, F334 and LEW rats were performed based on data and genome analysis tools at the Rat Genome Database (RGD), Rat Genome Database Web Site, Medical College of Wisconsin, Milwaukee, Wisconsin (http://rgd.mcw.edu/). Any gene within the region fine mapped using the above haplotype analysis that contained at least one non-synonymous SNP was identified using Ensembl's Biomart engine and the rat short variation (SNPs and indels) (Rnor_5.0) dataset. We then used the variant distribution tool on the RGD website to identify which SHR strain genes contained at least one SNP difference from F344 and BN strains. Nucleotide positions correspond to the RGSC3.4 assembly. Further fine mapping analyses were performed by whole transcriptome sequencing and novel SNP identification. RNA (Qiagen RNeasy Plus kit) was isolated from unprimed and LPS-primed (100 ng/ml) LEW and SD BMDMs or LPS-primed BN BMDMs. mRNA purified by polyA-tail enrichment (Dynabeads mRNA Purification Kit, Invitrogen) was fragmented into 200–400 bp, and reverse transcribed into cDNA before Illumina sequencing adapters (Illumina, San Diego, CA) were added to each end. Libraries were barcoded, multiplexed into 5 samples per sequencing lane in the Illumina HiSeq 2000, and sequenced from both ends (60 bp reads after discarding the barcodes). Sequences were mapped to the Rat genome (rn4) using Bowtie (2.0.2) [21] and Tophat (v2.0.4) [22]. To identify SNPs from the RNAseq data in the interval fine mapped above, Bam files were processed with samtools (0.1.16, r963:234) mpileup function, with rn4 as reference sequence. Read pileups were processed across all five samples using VarScan.v2.2.11 and the mpileup2snp function (parameters: –min-coverage 2 –min-reads2 1 –min-var-freq 0.01 –p-value 0.05 –variants). Resulting variant positions were annotated using UCSC Genome Browser's “Variant Annotation Integrator”. SNPs identified between 5 samples (2 SD, 2 LEW, 1 BN) were filtered for concordance and homozygosity between the two independent LEW samples and BN having the same nucleotide as the reference genome (which is from BN), and subsequently filtered for non-synonymous SNPs where LEW differed from BN and SD. It should be noted that not all known LEW SNPs in *Nlrp1* are discovered using this procedure as the N-terminal NLRP1 region contains a stretch of eight amino acids that differ between LEW and BN and our procedure for mapping reads to the genome does not allow for that many mismatches. Similar problems lead to underreported *Nlrp1* SNPs in the RGD website.

## Results

### NLRP1 sequence in inbred rats correlates with macrophage cell death, parasite proliferation and IL-1β/IL-18 release

**Figure 1:**
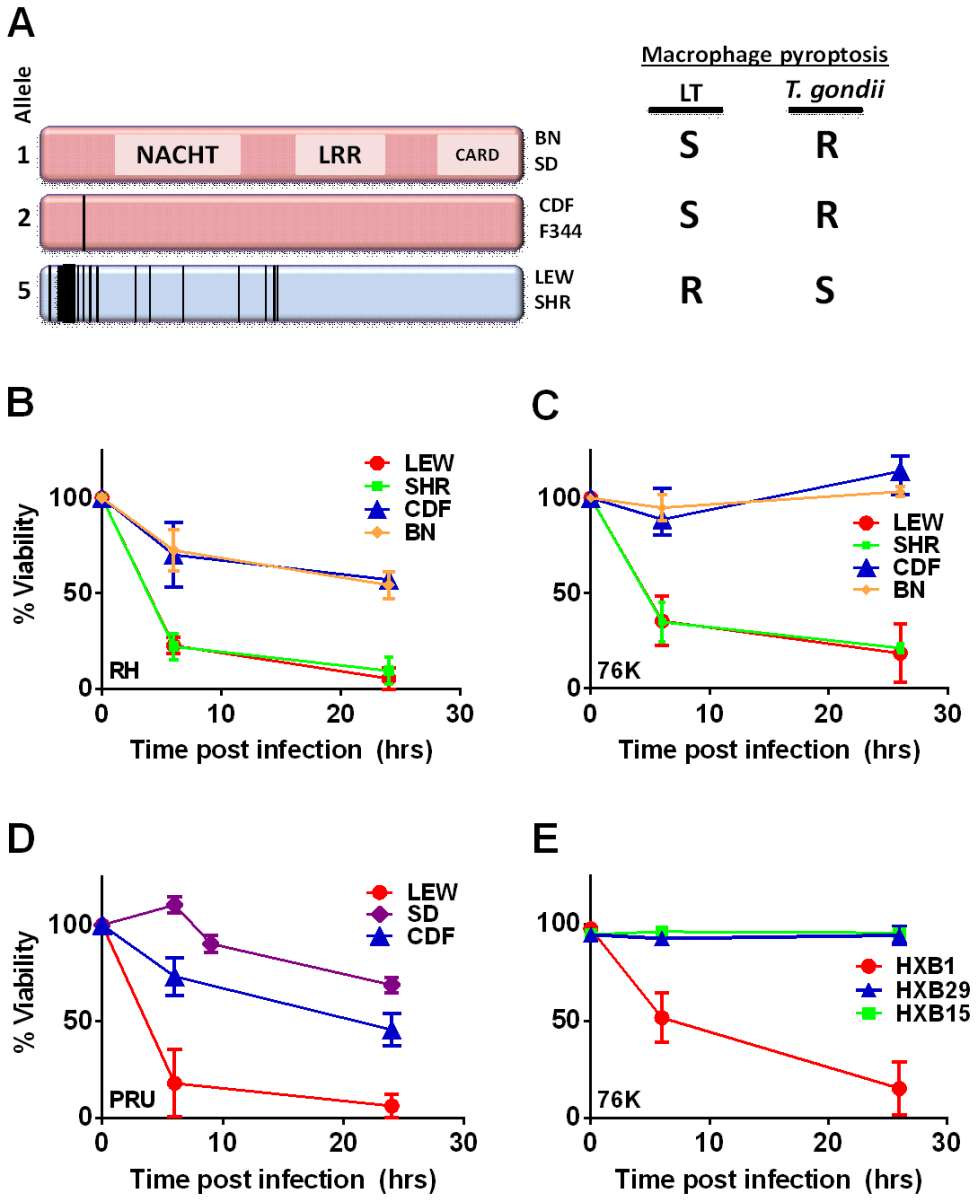
NLRP1 sequence in inbred and RI rats correlates with rapid macrophage death. (A) Sequence map of rat NLRP1 variants. This diagram was modified from [4]. Vertical black lines indicate amino acid polymorphisms relative to the protein encoded by allele 1. Approximate NACHT, LRR and CARD domain locations relative to polymorphisms are shown. Macrophage sensitivity to LT-induced pyroptosis for the listed rat strains is from [4] and *Toxoplasma* sensitivities are from this work. (B–E) Viability measurements for rat BMDMs from LEW, SHR (expressing NLRP1^variant5^); CDF, BN or SD (expressing NLRP1^variant 1,2^); or RI rat strains following infection with *Toxoplasma* Type I (RH) or Type II (76K or PRU) (MOI 3∶1) by MTT measurements. Data shown are average from three independent experiments with SD (triplicate wells/experiment/condition), except RI strains, which are averages from two experiments (triplicate wells/experiment/condition). Viability values were calculated relative to MTT measurements for uninfected control cells at each time point which were set at 100%. P-values comparing all NLRP1^variant 1,2^ -expressing strains to NLRP1^variant 5^ -expressing strains are <0.001.

The *Toxo1* locus on chromosome 10, which controls rat resistance to toxoplasmosis, maps within a region containing the inflammasome sensor *Nlrp1* gene. NLRP1 was previously shown to control rat macrophage sensitivity to pyroptosis by the anthrax protease LT. Sequencing of twelve inbred rat strains revealed five highly homologous variants, two encoding NLRP1 protein sensitive to LT-mediated cleavage activation (NLRP1^variant 1,2^), and three which encode LT-resistant proteins (NLRP1^variant 3,4,5^) (Figure 1A). We noted that rat strains encoding NLRP1^variant 1,2^ historically support parasite proliferation in myeloid cells while rat strains encoding NLRP1^variant 5^ do not. [2]. Therefore we investigated whether macrophages from rats expressing different NLRP1 variants also differed in inflammasome activation and pyroptosis upon parasite infection. Inflammasome activation was assessed by monitoring cell death and cleavage of pro-IL-1β (37 kD) with subsequent secretion of mature active IL-1β (17 kD). We infected BMDMs from LT-sensitive CDF, BN or SD (NLRP1^variant 1,2^) rat strains and LT-resistant LEW and SHR (NLRP1^variant 5^) rat strains with luciferase-expressing Type I (RH) and Type II (76K, or PRU) *Toxoplasma* strains at various MOIs. BMDM viability measurements showed that NLRP1^variant 5^-expressing macrophages underwent a rapid cell death after *Toxoplasma* infection starting at 3 h and completed by 24 h whereas the majority of the NLRP1^variant 1,2 −^-expressing macrophages remained viable and supported *Toxoplasma* growth even 24 h after infection (Figure 1B–D). The parasite itself did not contribute significantly to MTT or LDH signals (Figure S1, panels A, B) and DAMPs from lysed host cells also did not induce cell death (Figure S1 panels C, D). Results were unaltered when cells were pre-treated with LPS (100 ng/ml) prior and throughout infection (Figure S1 panels C, D). Fischer F344/NTac (NLRP1^ variant 2^) macrophages also showed resistance similar to that of Fischer CDF macrophages (data not shown). Both NLRP1^variant 1,2^ and NLRP1^variant 5^ -expressing macrophages were fully responsive to nigericin-induced NLRP3 activation (Figure S2 and [18]), indicating fully functional inflammasome assembly and caspase-1 function in these rat strains.

**Figure 2:**
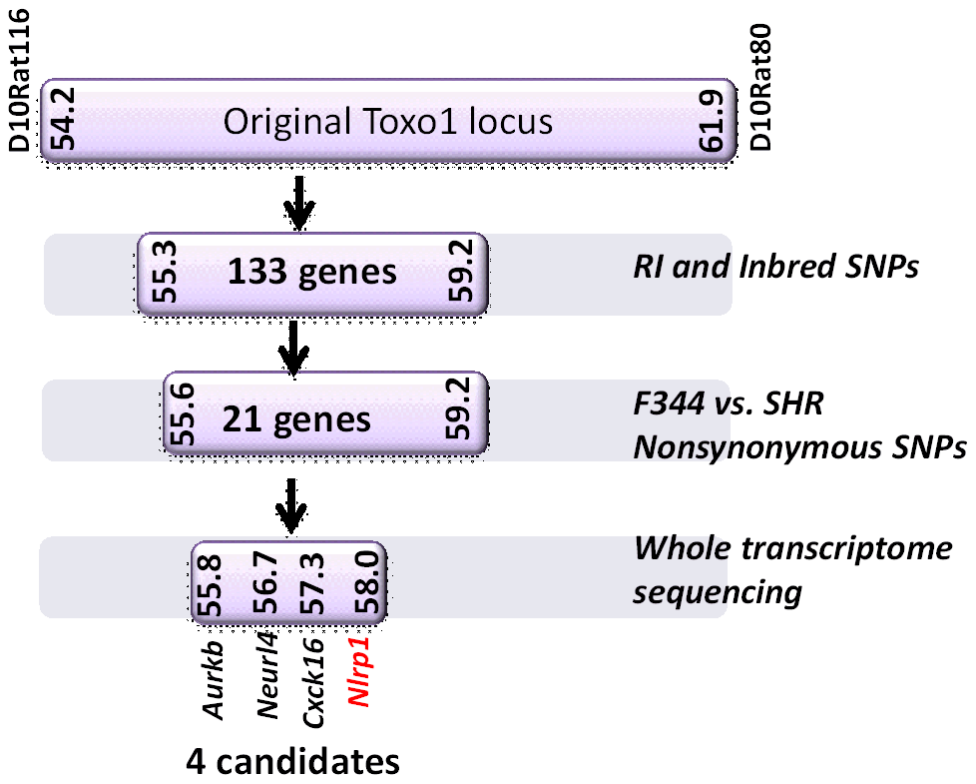
Summary flow diagram for mapping of rat macrophage sensitivity to four candidate genes. Methods for reducing the number of candidates at each stage are listed to the right and explained in detail in the Results section. Detailed SNPs and gene lists for each stage can be found in Supporting Figures S3 and S4 and Dataset S1.

We next tested macrophages from three rat strains (HXB1, HXB15 and HXB29) from the HXB/BXH recombinant inbred (RI) rat collection previously used to map LT sensitivity [4]. These strains have chromosome 10 crossover points closely flanking the *Nlrp1* locus, as indicated by SNP analyses. We found that macrophages from the RI strain HXB1, an LT-resistant strain, were sensitive to *Toxoplasma* Type I (RH) and Type II (76K) infection-induced lysis while the macrophages from the other two strains, which are LT-sensitive, were resistant to parasite induced rapid death (Figure 1E). These rats allowed us to reduce the *Toxo1* locus from the previous 54.2 Mbp–61.8 Mbp region to 54.2 Mbp–59.2 Mbp (Figure S3). We performed SNP and haplotype analyses for the CDF (F344/Crl), F344/NTac, BN (all strains with macrophages resistant to *Toxoplasma*-induced lysis) and the SHR strain (a strain with macrophages sensitive to *Toxoplasma*-induced lysis) and further narrowed the region determining resistance to 55.3–59.2 Mbp (between SNPs rs63997836 and rs106638778) (Figure S3). This region contained 133 genes of which 21 contained non-synonymous SNPs that were present in F334 and/or SHR rats, where genotype correlated with *Toxoplasma* resistance phenotype. To further narrow down the list of possible candidate genes, we performed whole transcriptome sequencing on BMDM from the LEW (pyroptosis-sensitive macrophages), BN (pyroptosis-resistant macrophages) and SD (pyroptosis-resistant macrophages) strains. We determined which genes were expressed in unstimulated and LPS-stimulated LEW BMDM (which are sensitive to parasite induced pyroptosis under both conditions), and contain SNPs that correlate with the resistance phenotype. Sixty-five of the 133 genes in the fine-mapped region were expressed (fragments per kilobase of transcript per million mapped reads >2) but only five of these contained non-synonymous SNPs that distinguished LEW from SD/BN (Dataset S1 and Figure S4). Although there were also differences in gene expression levels between LEW and SD/BN macrophages, none of the genes were expressed higher (1.5 fold) in both the non-stimulated and LPS stimulated LEW macrophages compared to the SD/BN macrophages (Dataset S1). By combining all analyses, we were able to narrow down the possible candidate genes to *Aurkb* (Aurora kinase B1, 55.7 Mbp, 1 SNP), *Neurl4* (neutralized homolog 4, 56.7 Mbp, 1 SNP), *Cxcl16* (chemokine C-X-C ligand 16, 57.3 Mbp, 1 SNP) and *Nlrp1* (6 SNPs). Figure 2 summarizes the above described mapping steps. Of these four genes, *Nlrp1* was the most likely candidate to be *Toxo1;* it contained the highest number of non-synonymous SNPs and is a known activator of the inflammasome. Our fine-mapping analyses combined with the established perfect correlation between sensitivity to *Toxoplasma* induced macrophage cell death and the NLRP1 N-terminal sequence in inbred and RI rats [4], which was in turn inversely correlated to rat resistance to chronic, transmissible *Toxoplasma* infection suggested that the *Toxo1* locus could be the *Nlrp1* gene.

**Figure 3:**
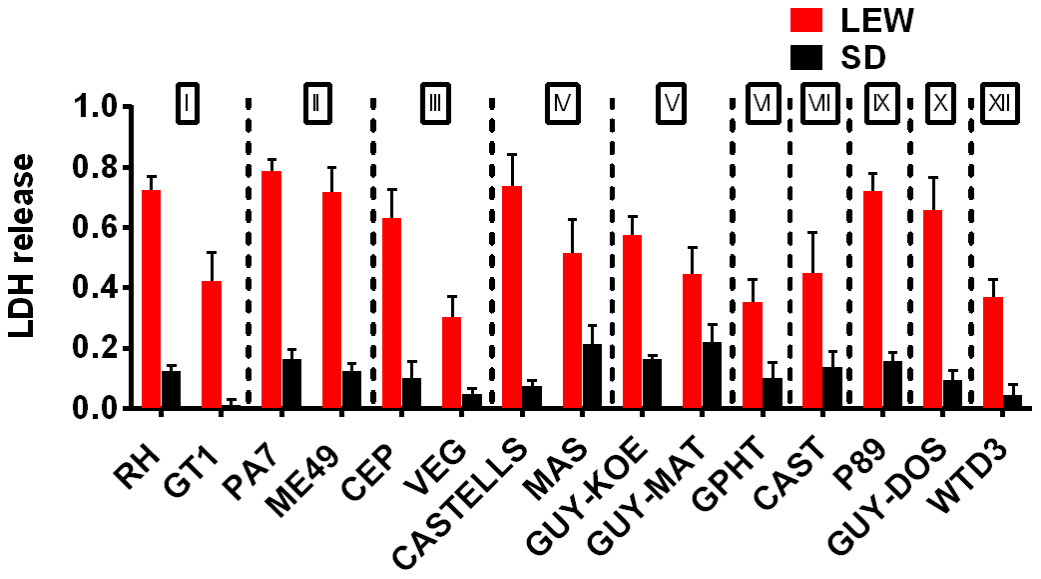
NLRP1 variant-dependent rapid cell death is induced by many different parasite strains. Viability as measured by LDH release for BMDMs from SD (NLRP1^1,2^ variant) or LEW (NLRP1^5^ variant) infected with strains representing global diversity for 24 h (Infection MOI 0.5–1 depending on strain, n = 4 wells/strain). P-values comparing LEW and SD <0.05 for all strains except MAS, CAST, GPHT and GUY-MAT.

**Figure 4:**
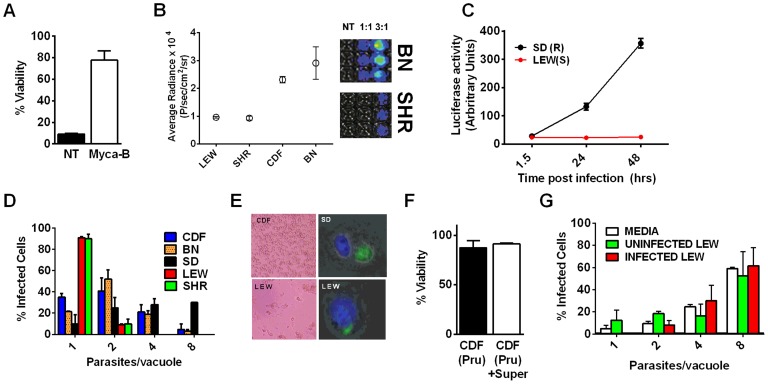
NLRP1-variant dependent macrophage death depends on parasite invasion and controls parasite proliferation. (A) Viability of LEW BMDMs infected with Mycalolide-treated (3 µM, 15 min) RH tachyzoites (MOI 1∶1) after 24 h as measured by MTS assay (P-value comparing Mycalolide group to untreated = 0.0002). (B, C) Radiance emission analyses of metabolically active, viable Type II *Toxoplasma* 76K parasites (B, graph MOI 3∶1, 6 h; inset shows representative plate from one experiment) or Type I RH parasites (C, MOI 1∶1 over 48 h) in BMDMs from different rat strains. P-value comparing NLRP1^variant 1,2^ expressing strains to NLRP1^variant 5^ expressing strains are <0.01 in I by t-test and <0.0001 in J by two-way ANOVA. (D) Number of parasites/vacuole in infected BMDMS (24 h, 3∶1 MOI) as assessed by microscopy is shown. CDF, BN infections were with 76K, and SD, LEW infections were with RH. Between 50–100 vacuoles counted per experiment. Average values from 3 experiments are shown for all strains, except SD (n = 2). P-values are <0.01 (two-way ANOVA) when comparing NLRP1^variant 1, 2^ expressing strains to NLRP1^variant 5^ expressing strains. (E) Left panels show light microscopy images of CDF and LEW monolayers infected with 76K (MOI 6∶1, 6 h). Right panels show fluorescence microscopy image of single SD and LEW BMDMs infected with RH (MOI 1∶1, 2 h). Blue is Hoechst stained nucleus, green are GFP-expressing parasites. Dividing parasites in SD cells (upper right) or a single parasite in LEW cells (lower right) are shown. (F) LEW BMDMs were infected with PRU (MOI 3;1) and at 5 h post infection culture supernatants from dying cells was spun, filtered and transferred to similarly infected (PRU, MOI 3∶1) CDF BMDMs. Viability of CDF BMDMs was assessed at 10 h post-infection by MTT staining. All values were calculated relative to uninfected control BMDMs (G) SD BMDMs were infected with RH parasites (2 h, MOI 1∶1), washed with PBS and medium replaced with fresh media, media from RH-infected (24 h, MOI 1∶1) or uninfected LEW BMDMs. Parasites/vacuole counted at 24 h. P-values >0.1 (ns) for comparison of any of three groups for 1, 2, 4 and 8 parasites/vacuole counts (by two-way ANOVA).

A survey of *Toxoplasma* strains that are genetically distinct from the archetypal I, II and III strains [23], [24] showed that they all induced NLRP1 variant-dependent rapid cell death (Figure 3). Because cell death was consistently dependent on MOI, we tested whether parasite invasion was required for cell death, as *Toxoplasma* can secrete effectors from its rhoptry organelle directly into the host cytoplasm. Parasites treated with Mycalolide B, a drug that blocks invasion but allows for secretion of microneme and rhoptry contents, attached but were unable to kill BMDMs, indicating that macrophage sensitivity to cell death was invasion-dependent (Figure 4A). Mycalolide B did not affect the viability of parasites or their ability to secrete rhoptry contents as verified by the observation that every cell with an attached mycalolide-B-treated parasite also had protein kinase ROP16 activation of STAT6 (Figure S5). Because *Toxoplasma* needs host cells for replication and the parasite replicates equally well in fibroblasts from different rat strains [2], we hypothesized that rapid macrophage cell death prevents *Toxoplasma* replication. We therefore investigated parasite proliferation in BMDMs from the different rat strains. *Toxoplasma* burden, as measured by bioluminescence, was significantly higher in infected NLRP1^variant 1,2^ -expressing BMDMs than NLRP1^variant 5^ -expressing cells (Figure 4B, 4C). This difference was independent of *Toxoplasma* strain but perfectly correlated with NLRP1 sequence and continued to increase over time only in the cell death-resistant BMDMs from *Toxoplasma* susceptible rat strains (Figure 4C). Similarly, GFP signal indicative of parasite load was higher in resistant cells from these rat strains (data not shown). Parasite proliferation was independent of LPS-priming (data not shown) and more parasites/vacuole were detected in NLRP1^variant 1,2^ -expressing macrophages compared to Nlrp1^variant 5^ -expressing cells (Figure 4D). Although only ∼10% of sensitive LEW (NLRP1^variant 5^) BMDMs were intact after 24 h of infection (Figure 4E left panels), 90% of these surviving cells contained single parasites (Figure 4E right panels). Nearly 100% of resistant SD, BN or CDF (NLRP1^variant 1,2^) BMDMs were intact after 24 h, and >60% of those infected contained multiple parasites per vacuole (Figure 4D, 4E). To determine if parasites released from lysed cells were viable, we measured the parasite's ability to reinvade macrophages by adding an antibody specific for the *Toxoplasma* surface protein, SAG1, to the medium of pre-infected BMDMs. We found that ∼35% of intracellular parasites in the sensitive LEW BMDMs were coated with the SAG1 antibody while only 5% were coated in resistant cells, demonstrating that some fraction of parasites released from rat BMDMs that rapidly lyse remain viable and capable of re-invasion (Figure S6). We verified that SAG-1 was not shed upon invasion by immunofluorescence, where 100% of parasites were stained for SAG1 when infected SD BMDMs were fixed and permeabilized at 18 h post-infection (Figure S6). Supernatants from lysed *Toxoplasma*-sensitive BMDMs also did not contribute to the rapid pyroptosis of resistant macrophages (Figure 4F) or alter parasite proliferation within these cells (Figure 4G).

**Figure 5:**
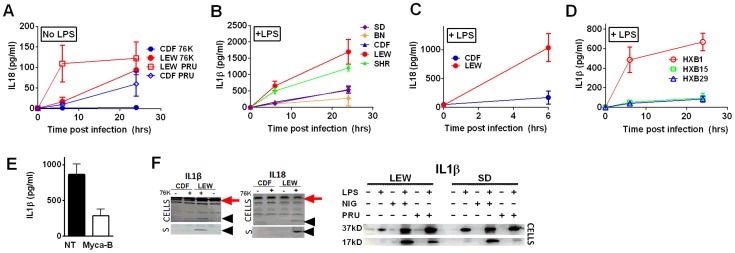
NLRP1-variant dependent cytokine cleavage and secretion. IL-18 (A, C) and IL-1β (B, D) from LPS-primed (0.1 µg/ml, 1 h) (B, C, D) or unprimed (A) rat BMDMs following *Toxoplasma* infection (MOI 3∶1 for 76K and 3∶1 and 5∶1 for PRU). All infections are with strain 76K unless otherwise indicated with the additional exception that SD BMDMs in panel B were infected with PRU. Results shown are averages from three experiments with SD shown, except measurements for PRU infections in panel A which are the averages of four experiments, two with MOI 3∶1 and two with MOI 5∶1 and those for the RI rats, which are from two independent experiments (triplicate wells/experiment/time point). No IL-1 of IL-18 release was measurable from uninfected controls at any time point for any of the experiments in A–D. P-values in (A) comparing CDF and LEW groups in (A) and (C) are <0.001 by two-way ANOVA. In (B) and (D), all P-values comparing NLRP1^ variant 5^ expressing strains to the NLRP1^ variant 1, 2^-expressing strains are <0.001 in all comparison combinations, by two-way ANOVA (E) IL-1β measurements from LPS-primed LEW BMDMs infected with Mycalolide-treated (3 µM, 15 min) RH tachyzoites (MOI 1∶1) after 24 h; P-value comparing Mycalolide group to untreated is 0.0024 (F) Western blot analyses for IL-18 and IL-1β in cell lysates and culture supernatants (indicated by “S”) of 76K-infected CDF and LEW BMDMs (4 h, MOI 3∶1)(left panels) or PRU infected LEW and SD BMDM cell lysates (MOI 3∶1, 24 h)(right panels). NLRP3 agonist nigericin (40 µM, 4 h) was used as a positive control for inflammasome activation in the gel shown on the right. In the left pair of gels, supernatants (no concentration, mixed 1∶1 with SDS loading buffer) were loaded and Westerns were visualized using IR-dye conjugated secondary antibodies and the LiCOR Odyssey. Cell lysates were also run, with processed IL-1β and IL-18 shown with arrowheads in these gels, and pro-forms shown by red arrow. In the right gel, cell lysates are shown in Westerns visualized by chemiluminescence using a charge-coupled device camera. The unprocessed form of IL-1β is shown as the 37-kD band, and the mature form is labeled 17 kD.

To investigate whether *Toxoplasma* infection induced maturation and secretion of IL-1β and IL-18 in an NLRP1 sequence-dependent manner, we measured secreted levels of these cytokines in the different rat strains. In the absence of LPS priming, Type II strain-infected BMDMs did not produce IL-1β (data not shown), but low levels of IL-18 were measurable by 6 h (PRU) and 24 h (76K) of infection in an NLRP1 variant-dependent manner. Thus in the unprimed situation, both 76K and PRU produced a much higher response in the LEW macrophages (expressing NLRP1^variant 5^) when compared to infection of CDF macrophages (expressing NLRP1^variant 2^) with the same Type II strain (Figure 5A). After LPS-priming, high levels of IL-1β and IL-18 secretion also correlated with NLRP1 sequence and macrophage sensitivity to rapid lysis (Figure 5B, 5C). Furthermore, the HXB1 (NLRP1^variant 5^), HXB15 and HXB29 (NLRP1^variant 1^) RI strains also produced IL-1β after infection in a manner correlated with NLRP1 sequence and macrophage sensitivity to *Toxoplasma* (Figure 5D). No IL-1β or IL-18 release was measurable from uninfected controls at any time point for any of the experiments shown in Figures 5A–D (data not shown). If parasites were treated with Mycalolide B, there was a significant reduction in cytokine production (Figure 5E) indicating that parasite invasion was necessary for inflammasome activation. Finally, cleavage of IL-1β and IL-18 was detected in cell lysates from LPS-primed, 76K or PRU-infected LEW, but not infected CDF and SD BMDMs, and cleavage correlated with cytokine secretion (Figure 5F). Nigericin activation of the NLRP3 inflammasome in both *Toxoplasma*-sensitive (LEW, NLRP1^variant 5^-expressing) and CDF or SD (NLRP1^variant1^-expressing) BMDMs confirmed previous findings that no general defect in the caspase-1 pathway was present in rats (Figure S1, 5F and [18]). Together these findings indicate a perfect correlation between sensitivity to *Toxoplasma-*induced macrophage cell death, decreased parasite proliferation, IL-1/IL-18 processing, rat resistance to *Toxoplasma* infection and NLRP1 sequence [4], suggesting that the *Toxo1* locus could be assigned to the *Nlrp1* gene.

### *Nlrp1* knockdown provides protection against *Toxoplasma-*induced pyroptosis

**Figure 6:**
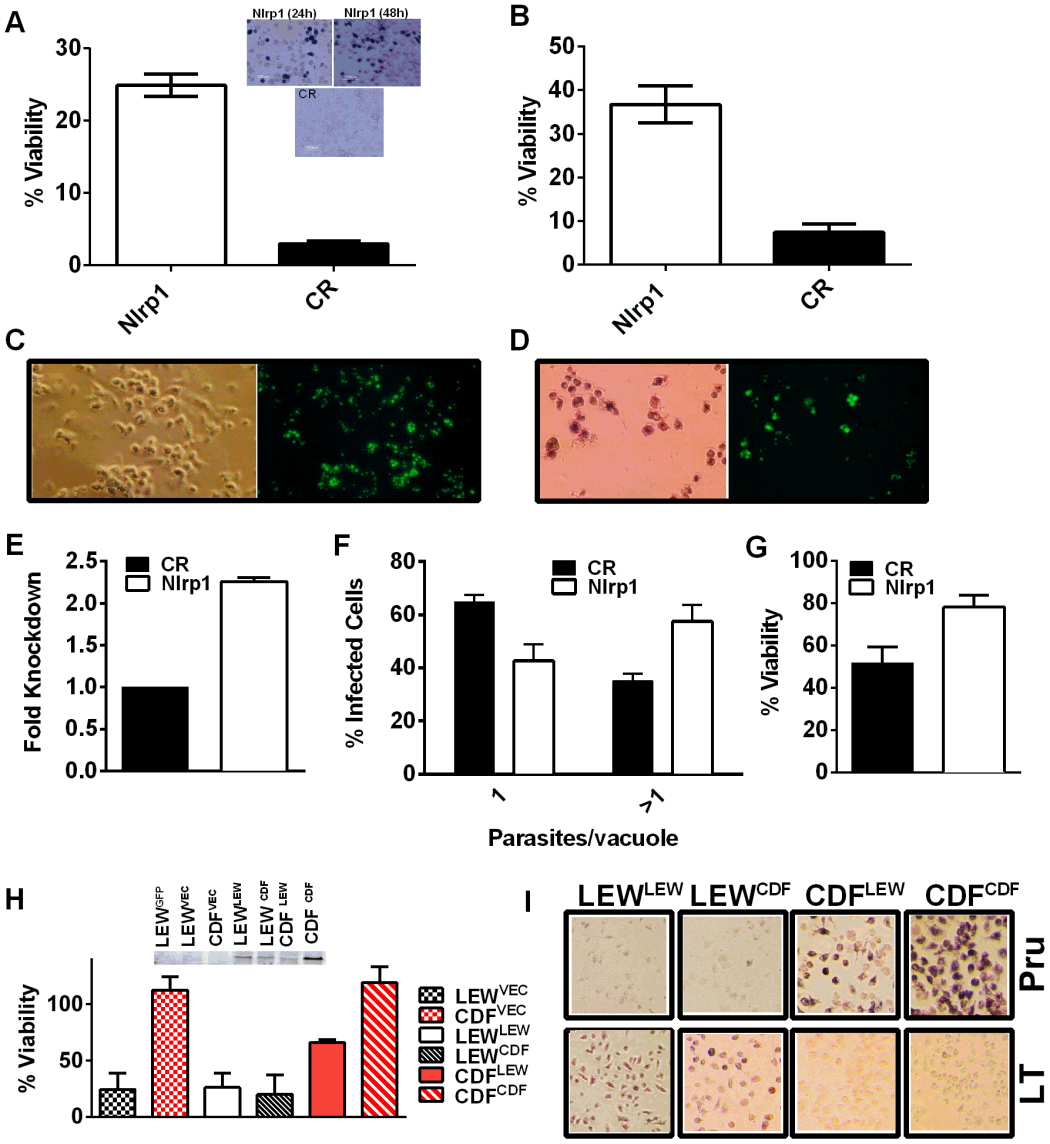
*Nlrp1* knockdown provides protection against *Toxoplasma-*induced pyroptosis and overexpression of NLRP1^variant 5^ sensitizes resistant macrophages. (A) Viability of LEW BMDMs nucleofected with *Nlrp1* siRNA pool or control siRNA (CR) 24 h or 48 h prior to infection with PRU (MOI 3∶1) as measured by MTT assay at 5 h post infection. Average from 6 separate nucleofection experiments (24 h n = 3, 48 h n = 3) are shown (triplicate wells/condition/experiment). P-values comparing *Nlrp1* siRNA to controls is <0.001. Microscopy images of MTT stained nucleofected cells from representative 24 h and 48 h knockdown experiments are also shown. (B) Viability of LEW BMDMs nucleofected with *Nlrp1* siRNA pool or control siRNA (CR) 36 h prior to infection with PRU (MOI 1∶1) as measured by MTT signal at 24 h post-infection. Average of 4 separate nucleofections are shown (triplicate wells/condition/nucleofection experiment) (C, D) *Toxoplasma* division in individually surviving nucleofected LEW BMDMs from (B) at 24 h post-infection. In C cells were fixed prior to microscopy, while in D cells were MTT-stained and fluorescence microscopy performed with no fixing. Note that all non-transfected or control siRNA transfected LEW macrophages which have succumbed are not present in these fields (detached by 24 h), while the MTT-negative ghosts and organelles of these lysed cells can be seen in parallel experiments at the earlier 5–6 h time, as shown in panel A. (E–G) Knockdown by the alternative lentiviral shRNA method was confirmed in LEW BMDMs by qPCR (E) and parasites per vacuole counts (F) and viability by MTS assay (G) were assessed in *Nlrp1-*knockdown LEW BMDMs after RH strain infection (MOI 0.5∶1). P-values by t-test comparing knockdown to controls is 0.03 for C and 0.01 for D. (H) Viability of LEW and CDF BMDMs nucleofected with full length HA-tagged NLRP1 constructs at −24 h prior to infection with PRU (MOI 5∶1) was measured by MTT assay at 5 h post-infection. Cell lysates from nucleofected cells were made at 32 h post-transfection and analyzed by Western using anti-HA antibody. Superscripts indicate the NLRP1 construct or vector that was transfected into the cell. Graph shows average from two nucleofection studies, with duplicate wells/condition/experiment. Lysates are from one of these nucleofections. There is no significant difference between any of the nucleofected LEW cells. The P-value comparing the CDF cells (expressing NLRP1^variant 2^) transfected with LEW (NLRP1^variant 5^) to CDF cells nucleofected with vector or CDF (NLRP1^variant 2^) is <0.0005. Presence of MTT-negative cells was also verified by microscopy for each well. Similar data is also shown in Figure S8, with anthrax LT control treatments. (I) Representative microscopy images of MTT viability staining for LEW and CDF BMDMs nucleofected with full length HA-tagged NLRP1 constructs −36 h prior to infection with PRU (MOI 3∶1) or treatment with LT (PA + LF, each at 1 µg/ml). MTT staining was performed on *Toxoplasma*-infected cells at 8 h post-infection and on LT-treated cells at 5 h post-infection. Superscripts indicate the NLRP1 construct or vector that was transfected into the cell.

We utilized two methods to knock down expression of rat *Nlrp1* (designated as *Nlrp1a* in the rat genome) to determine if NLRP1 mediates *Toxoplasma*-induced rat macrophage pyroptosis. First, an siRNA nucleofection approach was utilized. Only 20–35% of rat BMDMs can be transfected with this method, as assessed by control nucleofections with GFP expression vector and confirmed in parallel nucleofections in our current studies (data not shown). We found that there was a significant protection against LEW macrophage death in cells transfected with *Nlrp1* siRNA, compared to control siRNA, under conditions where 100% of BMDMs succumbed (Figure 6A and 6B). The 20–30% difference in viability was correlated with the number of successfully transfected cells, as reflected by the all-or-none nature of the protection in individual cells assessed by microscopy (Figure 6A, inset). Surviving LEW BMDMs remaining attached after longer periods of infection were verified to contain dividing GFP-expressing *Toxoplasma gondii* by fluorescence microscopy (Figure 6C, D), and viability was verified by MTT-staining (Figure 6D, left panel). Nonsurviving cells were completely detached from monolayers. A second method of knockdown by lentiviral delivery of a homologous mouse *Nlrp1b* shRNA was used to achieve a 2.2-fold reduction in *Nlrp1* expression compared to controls infected with a scrambled shRNA. Expression of *Nlrp1* was assessed by qPCR and standardized against actin levels (Figure 6E). Knockdown correlated with increased parasite proliferation and a higher number of vacuoles with more than one parasite (∼60%), compared to the macrophages treated with a scrambled control (35%) (Figure 6F). Host cell viability was also increased by 30% in the shRNA knockdown condition (Figure 6G).

### Overexpression of NLRP1^variant 5^ sensitizes CDF BMDMs, but not fibroblasts and mouse macrophages, to *Toxoplasma-*induced pyroptosis

We next overexpressed HA-tagged NRLP1^variant2^ and NLRP1^variant 5^ constructs [10] in rat BMDMs by nucleofection to test if this alters susceptibility to parasite-induced pyroptosis. The efficiency of transfection ranged from 25–40% in BMDMs in individual nucleofections (as assessed by monitoring of a co-transfected GFP construct in control cells). The LEW BMDMs did not gain resistance when transfected with the resistant CDF NLRP1^variant2^, but were sensitized to treatment with anthrax LT, confirming expression of the CDF NLRP1^variant2^ in a subpopulation of nucleofected cells (Figure S7). There was a significant sensitization to parasite-induced pyroptosis in CDF cells transfected with the LEW NLRP1^variant5^ (Figure 6H, Figure S7), while these cells remained almost 100% susceptible to rapid lysis by LT (Figure S8). Microscopy confirmed cell death for both *Toxoplasma*-infected CDF cells expressing LEW NLRP1^variant5^ and LT-treated LEW cells expressing the CDF NLRP1^variant2^ (Figure 6I). These results confirm that the LEW NLRP1^variant 2^ -mediated sensitivity to *Toxoplasma* is dominant, much in the manner the resistance of LEW rats to the parasite was previously shown to be a dominant trait [2]. They also re-confirm that the sensitivity to anthrax LT, mediated by the CDF NLRP1^variant2^ is a dominant trait. Interestingly, fibroblast HT1080 lines expressing these rat NLRP1 constructs [10] were not sensitized to *Toxoplasma-*induced pyroptosis even when transiently transfected and confirmed to express caspase-1 along with NLRP1 (Figure S8, panel A). These results confirmed that a macrophage cofactor or the macrophage cellular environment is required for parasite-induced pyroptosis. Furthermore, infection of mouse macrophage cell lines stably expressing rat NLRP1 constructs also did not result in sensitization to *Toxoplasma* (Figure S8, panel B), suggesting the presence of other factors in murine macrophages, or the BMAJ macrophage cell line, that result in a dominant resistance to pyroptosis or the absence of a factor needed for interaction with rat NLRP1 and subsequent pyroptosis. All tested mouse macrophages from any inbred strain, to date, have been resistant to *Toxoplasma*-induced pyroptosis (data not shown and Figure S8, panel C). The competition of endogenous murine NLRP1a and NLRP1b proteins for co-factors required for pyroptosis in the mouse macrophage may explain this resistance.

Together, the results presented in this work indicate that *Nlrp1* expression contributes to the ability of BMDMs from rats resistant to *Toxoplasma* infection to control parasite replication, most likely because of its role in mediating *Toxoplasma*-induced macrophage pyroptosis.

## Discussion

The *Toxo1* locus that controls rat susceptibility to toxoplasmosis [2] was previously mapped to a region of rat chromosome 10 containing the inflammasome sensor *Nlrp1*. In this work we identify *Toxoplasma* as a novel pathogen activator of the NLRP1 inflammasome. Until this work, anthrax LT was the only known activator of this inflammasome sensor [4], [10], [25]. We now demonstrate that like LT, rapid *Toxoplasma*-induced rat macrophage cell death is a pyroptotic event for which sensitivity correlates to NLRP1 sequence. Type I, Type II and a variety of genetically diverse *T. gondii* strains induce rapid pyroptosis in macrophages derived from inbred rats expressing NLRP1^variant 5^, while macrophages from BMDMs expressing NLRP1^variant 1,2^ are resistant to the parasite. This is the inverse of what is known for LT, where NLRP1^variant 1,2^ confers sensitivity [4]. In rats, macrophage sensitivity to *Toxoplasma-*induced cell death inversely correlates with whole animal resistance to infection. Rat strains historically susceptible to chronic *Toxoplasma* infection (e.g., CDF, BN, SD; NLRP1^variant 1,2^) have pyroptosis-resistant macrophages whereas resistant rats that cure infection (e.g., LEW, SHR; NLRP1^variant 5^) harbor macrophages that undergo parasite-induced pyroptosis. This suggests that the ability of the macrophage to allow parasite proliferation and possibly dissemination is linked to resistance to parasite-induced macrophage pyroptosis. Similar findings were previously described for mouse *Nlrp1b*-mediated control of anthrax infection. Mice resistant to *Bacillus anthracis* have macrophages expressing *Nlrp1b* variants which confer macrophage sensitivity to anthrax LT, and resistance is linked to the IL-1β response induced by toxin [26], [27]. The idea of control of parasite proliferation at the macrophage level is supported by findings that macrophages are among the first cell types to be infected when an animal ingests *Toxoplasma* cysts or oocysts [11], [12] and innate immune cells are used to traffic from the site of infection to distant sites such as the brain [28].

In parallel to the consequences for parasite proliferation after NLRP1 activation, the pro-inflammatory cytokines, IL-1β and IL-18, which are substrates of caspase-1, are cleaved and released following inflammasome activation. We demonstrate that these events only take place after infection of pyroptosis-sensitive macrophages in a manner correlating with NLRP1 sequence. It is possible that the release of these cytokines of the innate immune system could also play a role in controlling toxoplasmosis. IL-18 was at one time known as “IFN-inducing factor” and the role of IFN-γ in resistance to *Toxoplasma* is extensively documented (for review see [1], [29]). Treatment of resistant LEW rats with anti-IFN-γ antibodies does not reverse resistance but results in a much stronger antibody response, while anti-IFN-γ antibody treatment in susceptible rats causes an increase in parasite burden [3]. Altogether these findings suggest that IL-18, (through actions by IFN-γ) could be important for inhibition of *Toxoplasma* replication in rats, but that the cytokine's actions do not necessarily prevent parasite dissemination. On the other hand, it is important to note that as *Toxoplasma* can replicate and form cysts in many cell types that do not undergo pyroptosis, macrophage death may play a role strictly in dissemination. Thus, we suggest the combined consequences of inflammasome activation, macrophage cell death and IL-1/IL-18 secretion, on both dissemination and parasite proliferation, may ultimately result in resistance to *Toxoplasma*.

The only difference between the NLRP1 proteins from *Toxoplasma*-resistant and *Toxoplasma*-sensitive inbred strains is an 8 aa polymorphic region in the N-terminus of the protein, in a region of unknown function [4]. LT cleaves NLRP1^variant 1, 2^ proteins to activate this sensor and induce pyroptosis, while NLRP1^variant 5^ is resistant to cleavage [10]. How *Toxoplasma* activation of NLRP1 varies between rat strains based on an 8 aa sequence difference is unclear. The similar induction of pyroptosis we observed with numerous *Toxoplasma* strains suggests that the factor activating NLRP1 is unlikely to be parasite strain specific, or at least is conserved among multiple strains. One logical hypothesis is that the parasite-encoded effector molecule responsible for activation of NLRP1 is, like LT, a protease, but one which targets the LT-cleavage resistant sequence found in NLRP1^variant 5^. *Toxoplasma* secretes a large number of proteases [30]–[35]. It is unlikely that such a secreted protease could be derived from the rhoptries, because rhoptry secretion into the host cell was not sufficient to induce cell death. To date, we have been unable to observe any cleavage of NLRP1 in *Toxoplasma* infected fibroblasts which overexpress an HA-tagged variant of the protein (data not shown). It has also been recently shown that *Toxoplasma* can secrete effectors post invasion beyond the parasitophorous vacuole membrane [36] and these could be candidate effectors for NLRP1 activation. An alternative hypothesis to the parasite causing direct cleavage of NLRP1 is that the N-terminal polymorphic region of rat NLRP1 affects this protein's interaction with a different host ‘sensor’ acting as adaptor for the inflammasome, much in the manner described for the NLRC4/NAIP5/NAIP6 inflammasome recognition of flagellin [37], [38]. This unknown adaptor would interact with *Toxoplasma* or its effectors in all macrophages but may be limited by its ability to interact with the N-terminus of NLRP1^variant 1,2^ in rat BMDMs, or alternatively it could act as a direct inhibitor with specificity for these variants. The likelihood of a proteolytic activation of NLRP1 is also reduced when considering the finding that mouse ortholog NLRP1b proteins harbor an LT-cleavage site similar to rat proteins [25] but are highly resistant to *Toxoplasma*-induced pyroptosis in a manner independent of NLRP1b sequence or LT sensitivity (Figure S8). Furthermore, mouse macrophages could not be sensitized by rat NLRP1 overexpression. This finding was in contrast to the sensitization of the same cells to LT-mediated cell death [10], suggesting resistance of mouse macrophages to *Toxoplasma-*induced pyroptosis was dominant to any NLRP1-mediated effect, or (less likely) that co-factors required for parasite-mediated activation were only present in rat cells. Alternatively, the endogenous *Toxoplasma* non-responsive NLRP1a and NLRP1b proteins in mouse macrophages could compete in a dominant manner with expressed rat NLRP1 for co-factors required for pyroptosis. Interestingly, human NLRP1 does not contain an LT cleavage site in its N-terminus (for review see [39]). Instead human NLRP1 contains a pyrin domain required for association with the adaptor protein ASC [40], which does not appear to play a role in NLRP1-mediated rodent cell death [41], [42]. SNPs prevalent in this N-terminal region of human NLRP1 have been correlated with the severity of human congenital toxoplasmosis [14]. In those studies, knockdown of NLRP1 in human monocytic lines led to reduced cell viability after *Toxoplasma* infection, perhaps by allowing uncontrolled division of the parasite. Unlike our findings in rat cells, a protective role for human NLRP1 against macrophage death was suggested. It seems likely that the cell death observed in these human cell studies, which occurred over a period of days, differs from NLRP1-mediated rapid pyroptosis of rat cells, which occurs over a period of hours. Future studies are required to determine the mechanism of NLRP1 action in human cells.

In summary, we have established that *Toxoplasma gondii* is a new activator for the NLRP1 inflammasome. The identification of *T. gondii* as the second pathogen to activate the NLRP1 inflammasome raises the question whether this parasite activates the sensor via a novel mechanism, or whether proteolytic cleavage is required, in a manner similar to anthrax LT.

## Supporting Information

Figure S1 **Parasite-derived MTT signal and LDH levels.** (A) CDF BMDMs were infected PRU (MOI 1 or 3) and MTT assessed at 6 h post-infection relative to uninfected controls (B) RH parasites at shown MOI were lysed in the absence of cells using the same volume to lyse uninfected BMDM monolayer used in typical experiments and LDH levels measured (C, D) Primed or unprimed (LPS 100 ng/ml, 2 h) LEW BMDMs were infected with RH (MOI 0.5 or 1.0, as indicated) or treated with LEW macrophages or HFFs that had been syringe-lysed and prepared in parallel to parasites. The volume of cell lysates added to LEW BMDMs is equivalent to the volume of parasites added at the MOI indicated in parentheses. Viability and IL-1β release were then assessed 24 h post infection.
(PDF)

Figure S2 **Activation of the NLRP3 inflammasome by nigericin in CDF and LEW rats.** CDF or LEW BMDMs were pre-treated with LPS (1 µg/ml, 2 h) followed by either LT (1 µg/ml LF+1 µg/ml PA, 90 min) or nigericin (10 µM, 1 h). In a separate experiment, SD BMDMs were LPS treated (100 ng/ml, 2 h) and either infected with RH strain (MOI 0.5, 6 h or 8 h), or treated with nigericin (40 µM, 4 h). Supernatants were Amicon-concentrated prior to Western blotting. The unprocessed form of IL-1β is 37 kD. The mature cleaved form is 17 kD.
(PDF)

Figure S3 **Fine-mapping of the** ***Toxo1*** **region using whole transcriptome sequencing, SNP and haplotype analyses.** Table was generated using SNPlotyper tool at RGD. Alternative SNP annotations can be found at that site. Shaded area indicates the new boundaries for *Toxo1* locus based on comparison of the inbred and RI rat strain SNP genotypes for the 7 rat strains BN, F344 (CDF), LEW, SHR, HXB1, HXB15 and HXB59.
(PDF)

Figure S4 **Whole transcriptome analyses of LEW, SD and BN rats.** Summary of genes expressed in both LPS primed and unprimed conditions are shown for which non-synonymous SNPs (NS) existed. For each SNP, comparison of *Toxoplasma*-resistant and *Toxoplasma*-sensitive rat genotype correlation to phenotype was then used to narrow *Toxo1* to four candidates, in red.
(PDF)

Figure S5 **Parasites treated with Mycalolide B are able to secrete ROP16 and induce activation of pSTAT6.** HFFs were infected with GFP-expressing type I parasites that were pretreated with 3 µM Mycalolide B or vehicle control for 15 minutes. Cells were infected for four hours and then fixed with 3% formaldehyde, permeabilized with 100% ethanol and blocked. A rabbit antibody against human pSTAT6 was used as the primary antibody, followed by a goat- anti-rabbit antibody conjugated to Alexa Fluor 594. Green = Parasite, Blue/Pink = Hoechst, Red = p-STAT 6.
(PDF)

Figure S6 **Parasites released from lysed macrophages can reinvade other cells.** A) SD or LEW BMDMs were infected with GFP-expressing RH (2 h), washed three times with PBS and the media was replaced with fresh media containing rabbit anti-SAG1 antibody. After 24 h cells were fixed, permeabilized and stained with Alexa Fluor 594 goat anti-rabbit antibody. Parasites are green, while SAG1 is red. The quantification of SAG1-antibody coated parasites was performed with a minimum of 50 vacuole counts per condition from 3 experiments. (B) Parasites do not shed SAG1 upon invasion of SD BMDMs. Cells were infected with GFP-expressing RH for 18 h, cells were fixed, permeabilized and stained with a rabbit anti-SAG primary antibody followed by Alexa Fluor 594 goat anti-rabbit antibody. SAG1 was detected on 100% of parasites in any infected cells. Green = parasite, Red = SAG1, Blue = Hoechst.
(PDF)

Figure S7 **Overexpression of Nlrp1 variants confers sensitivity to** ***Toxoplasma*** **and LT.** Viability of LEW and CDF BMDMs nucleofected with full length HA-tagged NLRP1 constructs at −36 h prior to infection with PRU (MOI 1∶1) was measured by MTT assay at 8 h post-infection. Viability of similarly nucleofected cells was measured 5 h after treatment with anthrax LT (PA + LF, each at 1 µg/ml). Superscripts indicate the NLRP1 construct or vector that was transfected into the cell. Graph shows average from three independent nucleofections per condition.
(PDF)

Figure S8 **Viability of different cell lines and BMDMs overexpressing rat NLRP1 following infection with** ***Toxoplasma*** **.** (A) HT1080 fibroblast cells or (B) BMAJ mouse macrophage cell lines expressing full length HA-tagged NLRP1^variant 2^ (CDF sequence) or NLRP1^variant 5^ (LEW sequence) were tested for viability following *Toxoplasma* infection. Infections were with Type I (RH and Type II (76K) strains (MOI 5∶1) were performed and viability was assessed 24 h post-infection. Details on constructions of these lines can be found in [10]. In select experiments myc-tagged caspase-1 was also transfected 24 h prior to infection. Values graphed are mean ± SD, n = 3 wells/treatment. (C) Various mouse macrophage cell lines and BMDMs from mouse strains were tested for susceptibility to infection as described above. RAW264.7 cells were not tested with the RH strain. There is no statistical difference between any of the groups or treatments in these studies.
(PDF)

Dataset S1 **Raw data set from whole transcriptome analyses of LEW, SD, and BN rats.** Expression values (fragments per kilobase of transcript per million mapped reads = FPKM) of the genes in the fine-mapped locus are shown. Genes with FPKM>2 were considered expressed.
(XLS)
