# Supplementary figures and images for: Inflammasome Sensor NLRP1 Controls Rat Macrophage Susceptibility to Toxoplasma gondii
Source: PLoS Pathog. 2014 Mar 13;10(3):e1003927. doi: 10.1371/journal.ppat.1003927 (PMC3953412; doi:10.1371/journal.ppat.1003927)

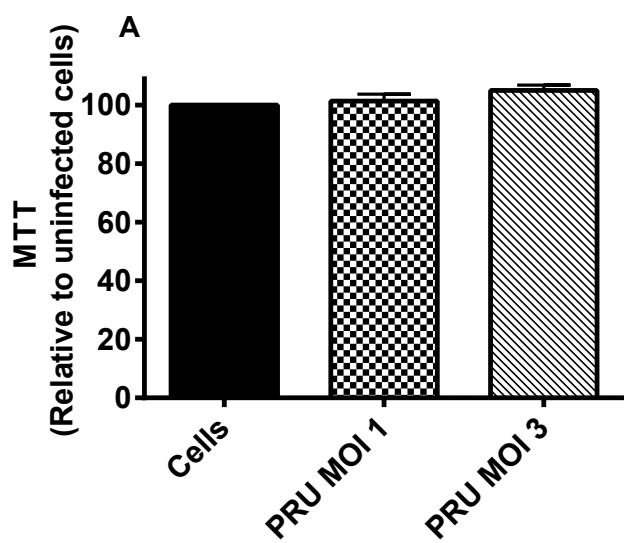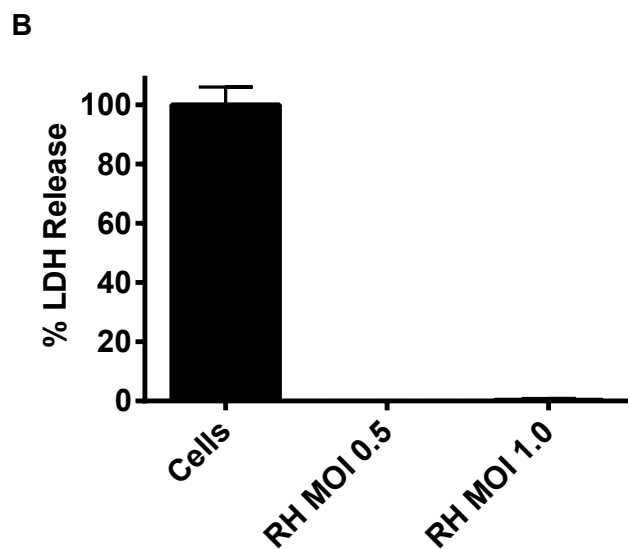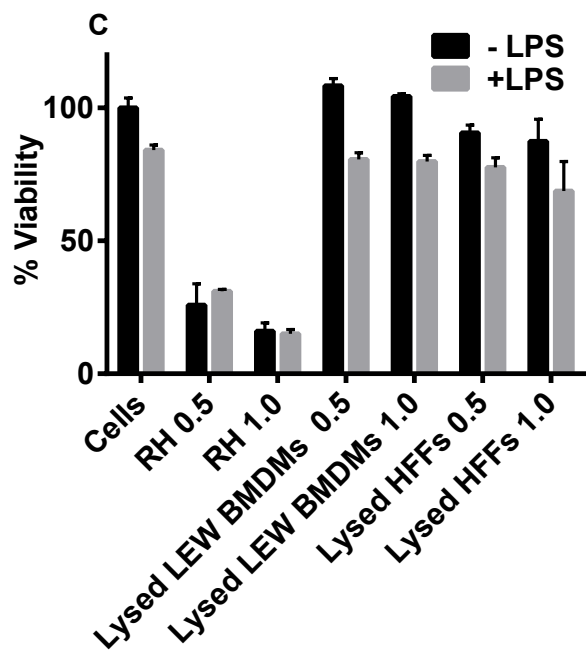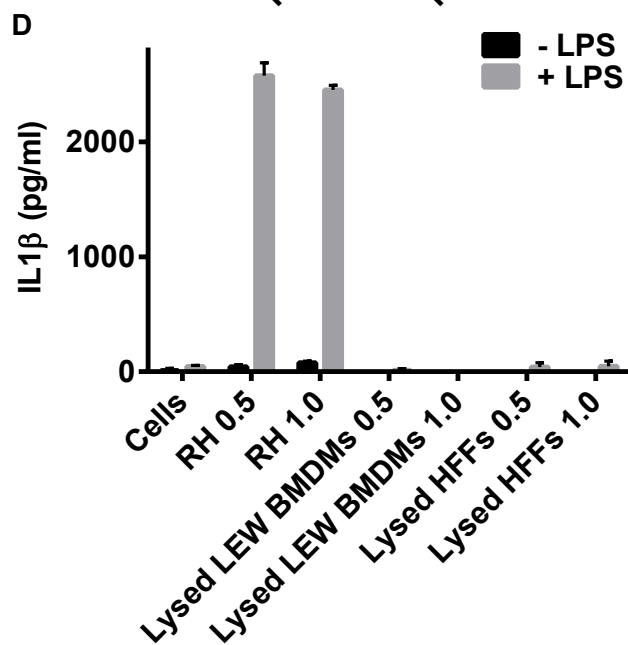

Supplement: Figure S1 — Parasite-derived MTT signal and LDH levels. (A) CDF BMDMs were infected PRU (MOI 1 or 3) and MTT assessed at 6 h post-infection relative to uninfected controls (B) RH parasites at shown MOI were lysed in the absence of cells using the same volume to lyse uninfected BMDM monolayer used in typical experiments and LDH levels measured (C, D) Primed or unprimed (LPS 100 ng/ml, 2 h) LEW BMDMs were infected with RH (MOI 0.5 or 1.0, as indicated) or treated with LEW macrophages or HFFs that had been syringe-lysed and prepared in parallel to parasites. The volume of cell lysates added to LEW BMDMs is equivalent to the volume of parasites added at the MOI indicated in parentheses. Viability and IL-1β release were then assessed 24 h post infection. (PDF) [file ppat.1003927.s001.pdf]

|             | <u>LEW</u> | <u>CDF</u> | <u>LEW</u> | <u>CDF</u> | <u>SD</u> | <u>SD</u> | <u>SD</u> |
|-------------|------------|------------|------------|------------|-----------|-----------|-----------|
| <b>LPS</b>  | +          | +          | +          | +          | +         | +         | +         |
| <b>LT</b>   | +          | +          | -          | -          | -         | -         | -         |
| <b>NIG</b>  | -          | -          | +          | +          | +         | -         | -         |
| <b>TOXO</b> | -          | -          | -          | -          | -         | +         | +         |

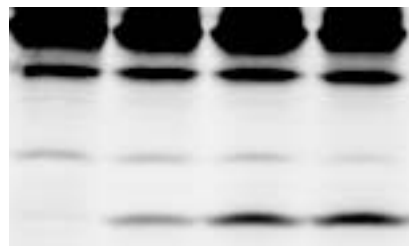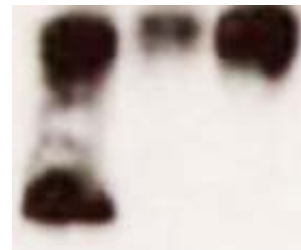

← 37kDa

← 17kDa

Figure S2

Supplement: Figure S2 — Activation of the NLRP3 inflammasome by nigericin in CDF and LEW rats. CDF or LEW BMDMs were pre-treated with LPS (1 µg/ml, 2 h) followed by either LT (1 µg/ml LF+1 µg/ml PA, 90 min) or nigericin (10 µM, 1 h). In a separate experiment, SD BMDMs were LPS treated (100 ng/ml, 2 h) and either infected with RH strain (MOI 0.5, 6 h or 8 h), or treated with nigericin (40 µM, 4 h). Supernatants were Amicon-concentrated prior to Western blotting. The unprocessed form of IL-1β is 37 kD. The mature cleaved form is 17 kD. (PDF) [file ppat.1003927.s002.pdf]

Toxo Hoechst pSTAT6

Untreated

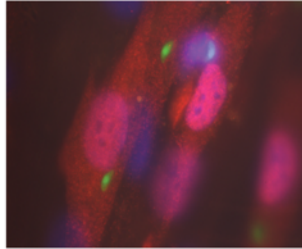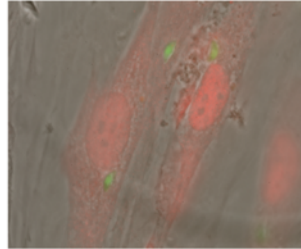

Mycalolide B

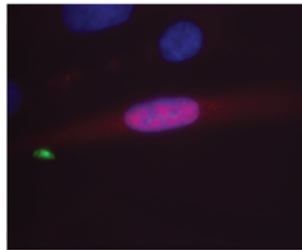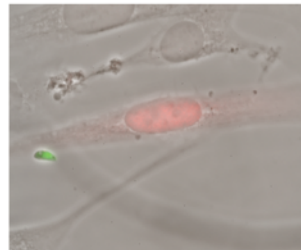

Supplement: Figure S5 — Parasites treated with Mycalolide B are able to secrete ROP16 and induce activation of pSTAT6. HFFs were infected with GFP-expressing type I parasites that were pretreated with 3 µM Mycalolide B or vehicle control for 15 minutes. Cells were infected for four hours and then fixed with 3% formaldehyde, permeabilized with 100% ethanol and blocked. A rabbit antibody against human pSTAT6 was used as the primary antibody, followed by a goat- anti-rabbit antibody conjugated to Alexa Fluor 594. Green = Parasite, Blue/Pink = Hoechst, Red = p-STAT 6. (PDF) [file ppat.1003927.s005.pdf]

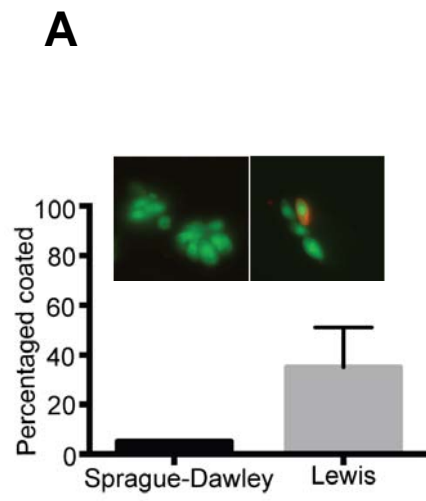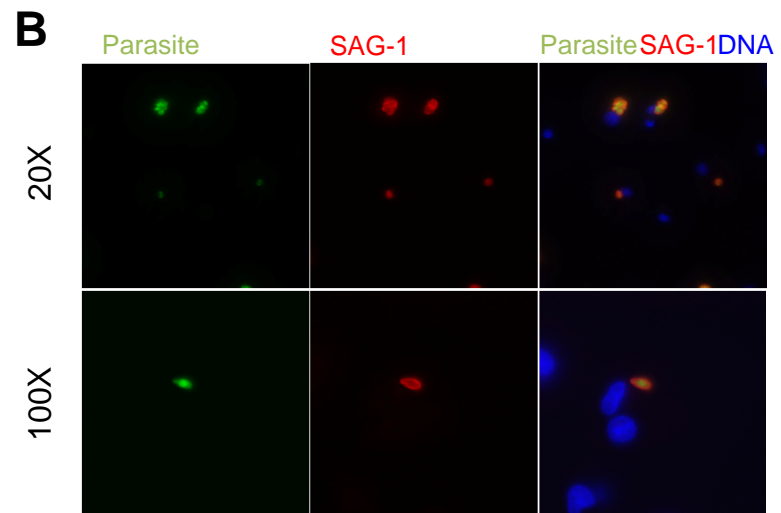

Supplement: Figure S6 — Parasites released from lysed macrophages can reinvade other cells. A) SD or LEW BMDMs were infected with GFP-expressing RH (2 h), washed three times with PBS and the media was replaced with fresh media containing rabbit anti-SAG1 antibody. After 24 h cells were fixed, permeabilized and stained with Alexa Fluor 594 goat anti-rabbit antibody. Parasites are green, while SAG1 is red. The quantification of SAG1-antibody coated parasites was performed with a minimum of 50 vacuole counts per condition from 3 experiments. (B) Parasites do not shed SAG1 upon invasion of SD BMDMs. Cells were infected with GFP-expressing RH for 18 h, cells were fixed, permeabilized and stained with a rabbit anti-SAG primary antibody followed by Alexa Fluor 594 goat anti-rabbit antibody. SAG1 was detected on 100% of parasites in any infected cells. Green = parasite, Red = SAG1, Blue = Hoechst. (PDF) [file ppat.1003927.s006.pdf]

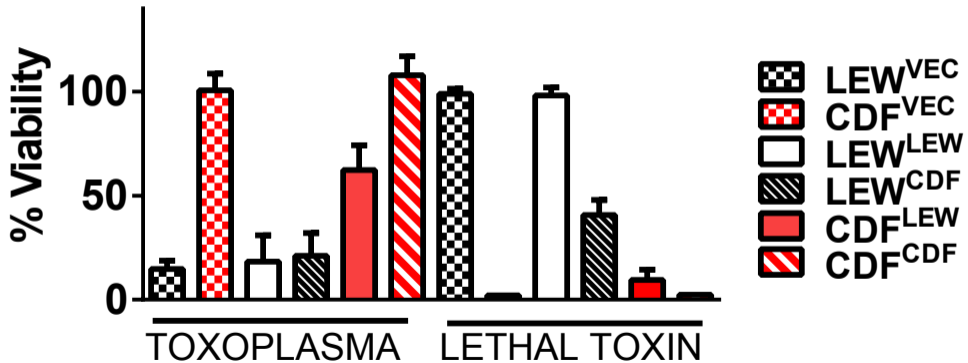

Supplement: Figure S7 — Overexpression of Nlrp1 variants confers sensitivity to Toxoplasma and LT. Viability of LEW and CDF BMDMs nucleofected with full length HA-tagged NLRP1 constructs at −36 h prior to infection with PRU (MOI 1∶1) was measured by MTT assay at 8 h post-infection. Viability of similarly nucleofected cells was measured 5 h after treatment with anthrax LT (PA + LF, each at 1 µg/ml). Superscripts indicate the NLRP1 construct or vector that was transfected into the cell. Graph shows average from three independent nucleofections per condition. (PDF) [file ppat.1003927.s007.pdf]

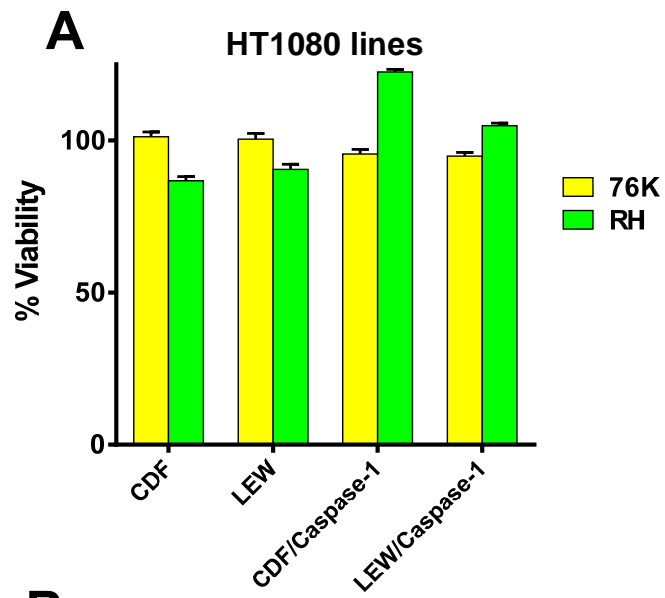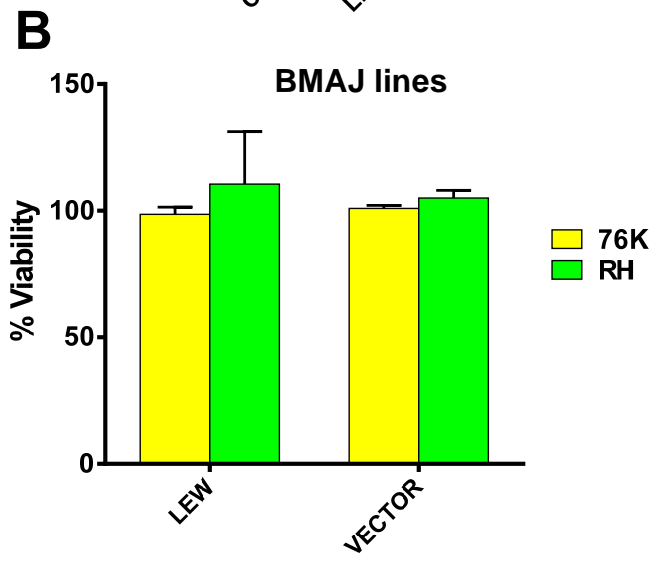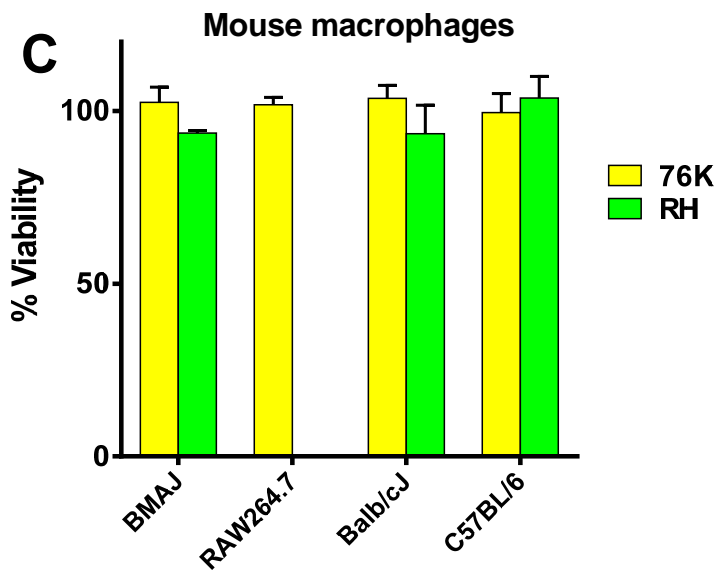

Supplement: Figure S8 — Viability of different cell lines and BMDMs overexpressing rat NLRP1 following infection with Toxoplasma . (A) HT1080 fibroblast cells or (B) BMAJ mouse macrophage cell lines expressing full length HA-tagged NLRP1variant 2 (CDF sequence) or NLRP1variant 5 (LEW sequence) were tested for viability following Toxoplasma infection. Infections were with Type I (RH and Type II (76K) strains (MOI 5∶1) were performed and viability was assessed 24 h post-infection. Details on constructions of these lines can be found in [10]. In select experiments myc-tagged caspase-1 was also transfected 24 h prior to infection. Values graphed are mean ± SD, n = 3 wells/treatment. (C) Various mouse macrophage cell lines and BMDMs from mouse strains were tested for susceptibility to infection as described above. RAW264.7 cells were not tested with the RH strain. There is no statistical difference between any of the groups or treatments in these studies. (PDF) [file ppat.1003927.s008.pdf]
