# Supplementary material for: Inflammasome Sensor NLRP1 Controls Rat Macrophage Susceptibility to Toxoplasma gondii
Source: PLoS Pathog. 2014 Mar 13;10(3):e1003927. doi: 10.1371/journal.ppat.1003927 (PMC3953412; doi:10.1371/journal.ppat.1003927)
Supplement: Figure S3 — Fine-mapping of the Toxo1 region using whole transcriptome sequencing, SNP and haplotype analyses. Table was generated using SNPlotyper tool at RGD. Alternative SNP annotations can be found at that site. Shaded area indicates the new boundaries for Toxo1 locus based on comparison of the inbred and RI rat strain SNP genotypes for the 7 rat strains BN, F344 (CDF), LEW, SHR, HXB1, HXB15 and HXB59. (PDF) [file ppat.1003927.s003.pdf]

			**Animal SENSITIVITY TO TOXOPLASMOSIS**	**S**	**S**	**S**	**R**	**S**	**R**	**R**
			**Macrophage SENSITIVITY TO PYROPTOSIS**	**R**	**R**	**R**	**S**	**R**	**S**	**S**
**SYMBOL**	**CHR 10 (Mbp)**	**ALLELE**	**SEQUENCE**	**BN**	**HXB15**	**HXB29**	**HXB1**	**F344**	**LEW**	**SHR**
ENSRNOSNP2796609	52519109	A/G	GCCCAGTTCTAAAGCTCATCTGAGTTTCCCATTGCCAGCT[A/G]CAACTGTGGCTACGCCAGTCAGGTTCTCATCCAAGAGAGC	AA	AA	AA	AA	AA	AA	GG
ENSRNOSNP2796612	53140716	G/A	ACCTGTTTCAAAAAAAAAAGAAAGAAAAGTAAGAAAAGAA[G/A]CAAAATGAAAAGAAAAAGGCTTACAATGAATCTTGTGTTT	GG	GG	GG	GG	AA	AA	AA
ENSRNOSNP2796615	53670113	T/C	CACAGTGACTTAATGGAGATTGGCTAGTTCTACACACCTC[T/C]GTTCCTCTGTTCAAACCTAAAACTCAGGTTAGGCCCTGAG	TT	TT	TT	TT	CC	CC	CC
ENSRNOSNP2796617	54102210	T/C	CTGGGATACATTGTAGCTCCTTTAAGGTTCACATTACCCC[T/C]AAGCATCTAGGAAGTTGAGGAGATGCAAGTAGGAAGTAAG	TT	TT	TT	TC	CC	CC	CC
ENSRNOSNP2796618	54201223	T/C	GTCTTGGTGACAGGCGTGGAGCCAGCTGTGGAAGACCCTG[T/C]AGCCTGGGACCAAGTTCCTGATGCTCTGCAGGAGTGCTGC	TT	TT	TT	CC	CC	CC	CC
D10Rat116	54220110		**Original Toxo1 boundary**							
ENSRNOSNP2796619	54318945	T/C	TACACTTCGTTCTGCTGCGACCTGCATTAATTATTTCTGC[T/C]AGGTTTGCACAGCAGGCCTGGGGCTGTCTGCTGCTGTACT	TT	TT	TT	NN	CC	CC	CC
ENSRNOSNP2796620	54332699	T/C	TCCATGTGAGTTGTTTAAGGGAATTTTCCTGGTGACTTGG[T/C]TGTGATTTTCTTTTCCTGCTTGAGTTGACTTCAGAAGGTC	TT	TT	TT	CC	TT	TT	CC
ENSRNOSNP2796622	54519676	A/G	TTAGATGTCATCGAGGGATCATGTCTCCCTACTGACAGGC[A/G]GGGTAGGGTCACAGTGAGTGTCTCCCTTGGGCCAGCCTTG	AA	AA	AA	GG	GG	GG	GG
ENSRNOSNP2796624	54684722	T/G	AAATAAATGGAGCATACCTGCATAGGGCATAAATTCACCA[T/G]TTTGAATTTGTGGCACACGTGTATAGTGTAATTCCACAGC	TT	TT	TT	GG	GG	GG	GG
ENSRNOSNP2796627	54778134	T/C	CAAGACACTTCTCTTTGTTTCCAAAGATAAAAGTAGCCTC[T/C]AAAAAGTTTTTTCCTATGCTGTACTTGGGAGTGGGTGACT	TT	TT	TT	NN	CC	CC	CC
ENSRNOSNP2796628	54956642	T/C	AGTCCCGATGGTAAAGCCAAGCATGGTGGCGAGAATATGC[T/C]GAACAGGATTGCTTAGTTCCTGACAGCCAGGAAGCAGAAA	TT	TT	TT	CC	CC	CC	CC
ENSRNOSNP2796631	55154737	T/C	GACTCTGGGCACAACTGGGGAAAAGACCCTTAAGTTGGAA[T/C]GAGGTTTGGGAAGGTATTAGTCAGGTGAAGAGAATAGAGA	TT	TT	TT	CC	CC	CC	CC
ENSRNOSNP2796632	55154923	C/T	AAGTGTCCAGCCTGTTCTAAGAGCTGCACCAGTACAGTGC[C/T]TCTAATGTATCATTTCAGAGAGGAGGAAACTGAGTCATAA	CC	CC	CC	TT	TT	TT	TT
ENSRNOSNP2796633	55303964	A/G	AGGATAGAAAAGGAGGTCAGAGCTGATGGGTTCAATATCT[A/G]TACAGAGTCCTGTGGCTGAGGAAGGAACAGGAAGTAGGTA	AA	AA	AA	GG	GG	GG	GG
ENSRNOSNP2796634	**55329725**	C/G	TAAAAGTTTTGGGGAGATGATCTCAGGTTGTTCCAGCTCT[C/G]AAATTCTGTGAGTCTACATGGTAAATGAAGAGGTGGCAGG	CC	CC	CC	GG	GG	GG	GG
			**New Toxo1 boundary**							
ENSRNOSNP2796635	55383531	A/G	CAGAGTGTACACAGTCCCAGACTTGAAAGAGGGCCAAGGT[A/G]GGGCTGTTTGTGGGAAACTGAGGAAGGTCTGGGGTAAGAT	AA	AA	AA	GG	AA	GG	GG
ENSRNOSNP2796636	55491552	T/C	AGGAAGCCCTGTTTCTTTGTGTACCCTGCTTCAGTCCCTG[T/C]CTCCAGGTTCTTGCCTTTGCTTGGCTCCTGCCTAGACTTC	TT	TT	TT	NN	TT	CC	CC
ENSRNOSNP2796637	55575040	A/G	CCCTGACCCTTCCACCACACCCGGTCTTAGAGCATTGTGA[A/G]CCCAACCTGCACTTGTCTCTCATTTCCTCTGAATAGTGAT	AA	AA	AA	GG	AA	GG	GG
ENSRNOSNP2796638	55581313	G/T	GTTTCCACATTAAAAGATTTTTTGGAGGGAAGTATCTTCT[G/T]TTAACCACATGATGATCCTATCCAGCTGCCCAGAGTCGGA	GG	GG	GG	TT	GG	TT	TT
ENSRNOSNP2796641	55622812	A/G	GCAATGACAGGGGTGAAAAGAAATGGAATGTTTCTGACCA[A/G]CCTGAGCTGATTGTATCACAGTCTCAGCCTAATTCCCACT	AA	AA	AA	NN	AA	GG	GG
ENSRNOSNP2796642	55717536	A/G	AAACAACTGAACGGGGTGGAGTGAACCCTACCCAAGCCTG[A/G]GAGGGAGGCTGCTGGGAGTTGCAGGGGCAGGTCCCTGTCA	AA	AA	AA	GG	GG	GG	GG
ENSRNOSNP2796643	55727468	C/T	TTTTCCTGCTTCTCCAGCTACCATTTCGCAACTCCGCGTT[C/T]TCCTAAAATACTATTTGGTCCCCCTAATTTGTCGTCCTAG	CC	CC	CC	TT	TT	TT	TT
ENSRNOSNP2796645	55815617	A/G	CAATGGTCATACCTCAAGTGCCGGGGGGCTTTAGGAACTT[A/G]AATGATCCCTGTCCACAACCATGCGAGGGCGAGGGTACAC	AA	AA	AA	GG	AA	GG	GG
ENSRNOSNP2796646	55985799	T/C	AAACAATTGCTTCCCGAGGTGTGGACAGAAAGGAGGACGT[T/C]GGGAACCAGTTCTACTTAGTCACGTGATGTTGTGGTCTGA	TT	TT	TT	CC	TT	CC	CC
ENSRNOSNP2796647	56049276	T/C	CTACCTCCCAACCAGCCATTTATGCTCCTTCCTCGACCAG[T/C]GGCCAGCGAGGACACTGACCAGAGGAAAGCTTTGCTAGTC	TT	TT	TT	CC	TT	CC	CC
ENSRNOSNP2796649	56118824	A/C	GCCCATCTCACTTGTTGCAATTTGAGCCCTTCCACCTATC[A/C]GTCTCCCAGAGCTTCAACCTGAAGGCCTACCACCCACTGA	AA	AA	AA	CC	AA	CC	CC
ENSRNOSNP2796651	56691083	C/G	CCTGAAGCAAAGCCTTCCTTGGCATACACTGCAGTGGACC[C/G]ACAAGGGTGACTGAGTGGTTGGGAGCCATTGTGTCCCATC	CC	CC	CC	GG	CC	GG	GG
ENSRNOSNP2796652	56691163	A/G	CCCTTCCCTCCTTCACCTGTGGCCCTGCAGAGGAGCATGT[A/G]TGAGACCCATTCTTCCTTGTGTTTACCATCTCCCAGCAGT	AA	AA	AA	GG	AA	GG	GG
ENSRNOSNP2796653	56691234	T/C	TCCCAGCAGTCCCTATGCACACATTAGCTTTTAAATTTCC[T/C]TGCCCACTCCTTCCAGCCTTCCTCCAGGGCCTCTGCACAG	TT	TT	TT	CC	TT	CC	CC
ENSRNOSNP2796656	56856324	G/A	GAAGGGGGCCTGTGTTTGGCATAGAAGAAGGCACACGACT[G/A]TGCTAGGGCCTGATTAGGAGTCTTCAGACTCTAAGGGGGT	GG	GG	GG	AA	GG	AA	AA
ENSRNOSNP2796657	57000555	C/T	TACTGCAGATTCCCAATGCCTTAGAGGTGTCCTATGCCCA[C/T]TTGCAGTGACAAGCCTTCACTGTCCCCCCAGCCCCCGTAG	CC	CC	CC	TT	CC	TT	TT
ENSRNOSNP2796659	57074418	A/G	GAAGTCAAAGGGTGAGGTGGTTATCAGGAACAGGGCGGGT[A/G]GCTCCAGCAGCCCTCTGACCCTCTCTCGTGCAGTCCTTGC	AA	AA	AA	GG	AA	GG	GG
ENSRNOSNP2796660	57077378	A/G	CTCGCCAGGGTAACAGTCCCAAAGGACACCAGATGAGTGG[A/G]ACAGATGGACTGAGGAGTCTTTAAATAAGGAGGGGAATGG	AA	AA	AA	GG	AA	GG	GG
ENSRNOSNP2796662	57261171	A/C	CAGAGGGTTGGGGCAAGAGGGTGGCAAGTGTGAGGTCAGC[A/C]TAGGATACGAAGCAAATCATGTTATAAACTGTAAGAATGA	AA	AA	AA	CC	AA	CC	CC
ENSRNOSNP2796663	57296249	T/C	TTGAACCTCGAACTTTACACTCCACCTCTGACTGCACTTT[T/C]GGACCACATACTTGAGAGTATTACATTTTATTAGAGGCAG	TT	TT	TT	CC	TT	CC	CC
ENSRNOSNP2796664	57302933	A/G	TTTCCGCAGAGGCTGCCCCCCTGCACCTCTCCTGCCTTTT[A/G]CTGGTGACGGATGAGCATGGCACCATCTTGGGCATTGATC	AA	AA	AA	GG	AA	GG	GG
ENSRNOSNP2796667	57733535	A/G	TAGTACGCATGCCTAAAAGCACCTGAGGCAGCCTCAGTGC[A/G]TCACTTCCGGCTGACCCTGGGGCTTGTCACTTGGTGGAAA	AA	AA	AA	GG	GG	GG	GG
ENSRNOSNP2796668	57841240	T/G	TCTGTGTGCTGGGCCGTGTGGGGATGGGCACCTCAGCCTG[T/G]CGCGGTCAGTCTGACTGCTGCACAGCTAGTGTGCATACAC	TT	TT	TT	GG	GG	GG	GG
ENSRNOSNP2796669	57846880	G/A	AGGGAACACTAATGAGACACTGGACTCGATTCCAACAGGC[G/A]TGGGATCAGATTGGGTGCCAGAAAAGTGCTGTTTCCTTGC	GG	GG	GG	AA	GG	AA	AA
ENSRNOSNP2796670	57875789	T/C	GAAACAGCCGGCACAAGCTCCTGAACAGGTACCGCCAGGC[T/C]GCAGGTAGCACACTGGGGACAGCCTCAGACAGACTTCTTG	TT	TT	TT	CC	CC	CC	CC
ENSRNOSNP2796671	57881403	G/A	AGGCTGGACAAGCCCCAAGCTGTCAGGTTTTGCTTTCTGA[G/A]GGTACTTGGTCCTAGCTTATAAGCTACACTGTTGATGGCG	GG	GG	GG	AA	GG	AA	AA
ENSRNOSNP2796673	58078553	G/T	CAAGATAAAGCCCATAGAAACAAAGTAGACATCTGATTAC[G/T]AGTGTAGAGGACAGGAGGAAATGGAAGATGCCGTTCCTC	GG	GG	GG	TT	GG	TT	TT
ENSRNOSNP2796674	58178197	T/A	CAGTAGATGAAGGTATAAAATATCAGAGGCATGTGTGTAT[T/A]AACTAAAGGGTAGAGACAGGGAGTGGCTTGGGGAAAATGG	TT	TT	TT	AA	TT	AA	AA
ENSRNOSNP2796675	58207771	G/A	CTCTCATCTCCTTTATTCTTTAGGGGTATCACTAAGCAGC[G/A]TAGGCTGGCTTTAAGCTTATTCTGTAGGCTGGGAAGACCT	GG	GG	GG	GG	AA	GG	GG
ENSRNOSNP2796676	58403857	C/T	CTACACATCTTCTCTCCACCTCACTCCATTTCTTCCATTA[C/T]AGTGCCACTGAAAAACACACAAAATAATTCAGAGTCATTT	CC	CC	CC	CT	TT	TT	TT
ENSRNOSNP2796677	58580166	C/T	GAGGGAAGACCCCGTGAAGGAAGTGAGAAGCTGAGGCATG[C/T]GATAGCAGACAAGGGACAAGTGCAGCAATGGGGGTGTGTG	CC	CC	CC	CT	CC	TT	TT
ENSRNOSNP2796679	58644488	C/T	CGCTTCGGGAGATGGTAGCCTTCCTCAATGTGTCCGTGAG[C/T]GAGGAGCGGCTTCTCTGTGTGGAGAACAACAAAGAGGGCA	CC	CC	CC	CT	CC	TT	TT
			**New Toxo1 boundary**							
ENSRNOSNP2796683	59247028	A/C	TTGTAGCTAGCCCCCAGTAGCTTCATCAGGGTTGTTAGGT[A/C]ACAGCCATACTAAGCATGTTATCTTACCAAGTTTCTTGTT	AA	AA	CC	NN	AA	CC	CC
ENSRNOSNP2796684	59585017	G/C	CTGTGTTCACTTTTTCTCACGTGGTTTACTCATCCACAGC[G/C]TCTTAAGGTTTTCCCAGTCTCTTTAAAGCAAACCCGGTCT	GG	GG	CC	GC	CC	CC	CC
ENSRNOSNP2796685	59591479	T/C	TGTGAGCCAAAATAAAGTCTTTCTCATTTAGTTGCTTTTG[T/C]AAAGAATTTTTCTCTTCGTAATAAGGACAGTGGCAGGACA	TT	TT	CC	TC	CC	CC	CC
ENSRNOSNP2796687	59686974	A/T	CTACCTCTGCCCCCCAAGCACTGGGATCAAAGGTGTGTCC[A/T]ACCACAATTATAATCATGTCACATATATGACAGAAATTAA	AA	AA	TT	AT	TT	TT	TT
ENSRNOSNP2796688	59894217	C/A	CTTCTCCCCACCTACAACTTCTCCCCGCCCACACACACAC[C/A]CCCAAATTAGCTTACTGCTTTGGGATTCAGCCTCATCCAC	CC	CC	AA	CA	AA	AA	AA
ENSRNOSNP2796689	59923802	A/G	ACAATACAGTCCTCACAAGCCAAGCATCAGTCCTAGGAGC[A/G]GGGATTCCCTGGTCGAGGCTGGACCTAGGCATAGGCTGCG	AA	AA	GG	AG	GG	GG	GG
ENSRNOSNP2796690	60056457	C/T	AGAACAGGTCCTGAGGGTTGGAGTGATGGTATAATTAGGC[C/T]GACACCCCTTCAGTGCCATGTCACTTTCTGGTCCTGGCTG	CC	CC	TT	NN	CC	TT	TT
ENSRNOSNP2796691	60099652	T/C	TTGCTGTGTGCTTCAGGAAACGTGCTGCACCATGGTTACC[T/C]CGAGTCCTCTCAACACAACCGGCTTAAAGGCACGTCCCAG	TT	TT	CC	TC	CC	CC	CC
ENSRNOSNP2796695	60454159	C/T	TCTTTCCTTCTACATGTACCTTTTTGTAATTTTTCTTTTA[C/T]AGTTCTAAAATTTTTGTGTTTTTGACTCCATTGTTTACAT	CC	CC	TT	CT	TT	TT	TT
ENSRNOSNP2796698	60882199	T/A	TTCTGTGTAGACTGTCTCCTTTTCCCAGTTGTTCCCAGCA[T/A]CAATGGCTTAAAGTCTCCAAAGCTTAGAAGCATTTGAGAA	TT	TT	AA	TA	AA	AA	AA
ENSRNOSNP2796701	61541376	A/G	GCTTGGCTGTTCTATAGCCTCCTTCTTCCTTCTCAAAGAC[A/G]TTCAACTTGCCTAACTCTCAGACTCGTGGGGTGATATGTT	AA	AA	GG	AG	GG	GG	GG
ENSRNOSNP2796702	61581419	G/C	TGCTAGAACCTATAATAAATTGTTAGTTTCCTTTTATGAC[G/C]TTTGGTTAATGGTTTTGCAATCTTTTGGAATGTGCTCTGA	GG	GG	CC	GC	CC	CC	CC
ENSRNOSNP2796704	61724019	C/A	CATTTTTACTTCGATGATAAACGATAATATGGGTTAAAAG[C/A]GTTCTTGTTTGCTGTTACAACACTTTAAAGATAGTATCCC	CC	CC	AA	CA	AA	AA	AA
D10Rat80	61798249		**Original Toxo1 boundary**							
