# Supplementary material for: Inflammasome Sensor NLRP1 Controls Rat Macrophage Susceptibility to Toxoplasma gondii
Source: PLoS Pathog. 2014 Mar 13;10(3):e1003927. doi: 10.1371/journal.ppat.1003927 (PMC3953412; doi:10.1371/journal.ppat.1003927)
Supplement: Figure S4 — Whole transcriptome analyses of LEW, SD and BN rats. Summary of genes expressed in both LPS primed and unprimed conditions are shown for which non-synonymous SNPs (NS) existed. For each SNP, comparison of Toxoplasma-resistant and Toxoplasma-sensitive rat genotype correlation to phenotype was then used to narrow Toxo1 to four candidates, in red. (PDF) [file ppat.1003927.s004.pdf]

	Subset of genes expressed in LEW BMDMs (+/- LPS) with non-synonymous SNPs									
**GENE**	**Start**	**End**	**LEWIS-NS**	**LEWIS-LPS**	**SD-NS**	**SD-LPS**	**BN-LPS**	**NS SNP (All rats) (Ensembl)**	**Different in SHR vs F334/BN (RGD)**	**Different in SD/BN vs. LEWIS (RNAseq)**
**Aurkb**	55798709	55804367	20.4	4.2	15.8	9.4	11.8		**YES**	**YES**
**Neurl4**	56745821	56757435	5.1	2.5	4.0	3.6	3.4	**YES**	**YES**	**YES**
**Cxcl16**	57296125	57307217	100.5	264.3	111.6	368.1	262.9	**YES**	**YES**	**YES**
**Nlrp1**	57963707	58007925	4.5	9.8	4.8	13.5	13.0	**YES**	**YES**	**YES**
Tp53	56399720	56411150	36.9	45.3	38.1	60.9	59.5	**YES**	**YES**	**NO**
Dlg4	56864458	56890626	2.3	3.1	2.2	2.9	2.3	**YES**	**YES**	**NO**
Arrb2	57244070	57284166	23.0	6.5	24.1	8.7	10.0	**YES**	**YES**	**NO**
**Rabep1**	57746746	57847498	9.5	18.1	7.6	12.0	11.1	**YES**	**NO**	**YES**
Rpl26	55662219	55665752	42.8	18.5	57.1	30.0	33.5	**YES**	**NO**	
RGD1563106	55768181	55788836	4.8	2.5	5.4	2.2	2.4	**YES**	**NO**	
Trappc1	56117228	56118815	68.7	66.7	57.4	56.9	62.5	**YES**	**NO**	
Lsmd1	56190118	56192730	50.7	27.4	45.0	26.6	33.8	**YES**	**NO**	
Eif4a1	56484231	56489739	348.4	422.1	335.2	312.3	307.2	**YES**	**NO**	
Rnasek	57077235	57078954	469.0	569.9	502.9	514.6	490.9	**YES**		
Psmb6	57370396	57372704	34.4	25.6	35.8	27.8	24.6	**YES**		
Pfn1	57531660	57534366	1123.6	1085.2	1133.8	1077.0	1134.6	**YES**		
Kif1c	57580645	57622517	30.8	31.0	27.8	24.3	19.6	**YES**		
C1qbp	57881782	57886434	113.0	72.1	96.4	87.7	97.2	**YES**		
Txndc17	59100037	59103010	135.1	107.9	165.1	76.9	81.8	**YES**		
XAF1	59185149	59196549	8.4	248.9	7.4	252.9	216.8	**YES**		
